# Array of metabolic pathways in a kleptoplastidic foraminiferan protist supports chemoautotrophy in dark, euxinic seafloor sediments

**DOI:** 10.1093/ismejo/wrae248

**Published:** 2024-12-13

**Authors:** Fatma Gomaa, Daniel R Rogers, Daniel R Utter, Christopher Powers, I-Ting Huang, David J Beaudoin, Ying Zhang, Colleen Cavanaugh, Virginia P Edgcomb, Joan M Bernhard

**Affiliations:** Department of Geology and Geophysics, Woods Hole Oceanographic Institution, Woods Hole, MA 02543, United States; Department of Organismic and Evolutionary Biology, Harvard University, Cambridge, MA 02138, United States; Chemistry Department, Stonehill College, Easton, MA 02357 United States; Division of Geological and Planetary Sciences, California Institute of Technology, Pasadena, CA 91125, United States; Department of Cell and Molecular Biology, College of the Environment and Life Sciences, University of Rhode Island, Kingston, RI 02881, United States; Department of Organismic and Evolutionary Biology, Harvard University, Cambridge, MA 02138, United States; Department of Biology, Woods Hole Oceanographic Institution, Woods Hole, MA 02543, United States; Department of Cell and Molecular Biology, College of the Environment and Life Sciences, University of Rhode Island, Kingston, RI 02881, United States; Department of Organismic and Evolutionary Biology, Harvard University, Cambridge, MA 02138, United States; Department of Geology and Geophysics, Woods Hole Oceanographic Institution, Woods Hole, MA 02543, United States; Department of Geology and Geophysics, Woods Hole Oceanographic Institution, Woods Hole, MA 02543, United States

**Keywords:** chemosynthesis, anaerobic metabolism, inorganic carbon uptake, dark carbon fixation, aphotic chloroplast sequestration, proton-pumping pyrophosphatases, microbial mat, *Beggiatoa*, Santa Barbara Basin, seafloor preservation

## Abstract

Investigations of the metabolic capabilities of anaerobic protists advances our understanding of the evolution of eukaryotic life on Earth and for uncovering analogous extraterrestrial complex microbial life. Certain species of foraminiferan protists live in environments analogous to early Earth conditions when eukaryotes evolved, including sulfidic, anoxic and hypoxic sediment porewaters. Foraminifera are known to form symbioses as well as to harbor organelles from other eukaryotes (chloroplasts), possibly bolstering the host’s independence from oxygen. The full extent of foraminiferal physiological capabilities is not fully understood. To date, evidence for foraminiferal anaerobiosis was gleaned from specimens first subjected to stresses associated with removal from in situ conditions. Here, we report comprehensive gene expression analysis of benthic foraminiferal populations preserved in situ on the euxinic (anoxic and sulfidic) bathyal seafloor, thus avoiding environmental alterations associated with sample recovery, including pressure reduction, sunlight exposure, warming, and oxygenation. Metatranscriptomics, metagenome-assembled genomes, and measurements of substrate uptake were used to study the kleptoplastidic foraminifer *Nonionella stella* inhabiting sulfur-oxidizing bacterial mats of the Santa Barbara Basin, off California. We show *N. stella* energy generation under dark euxinia is unusual because it orchestrates complex metabolic pathways for ATP production and carbon fixation through the Calvin cycle. These pathways include extended glycolysis, anaerobic fermentation, sulfide oxidation, and the presence of a membrane-bound inorganic pyrophosphatase, an enzyme that hydrolyzes inorganic pyrophosphate to actively pump protons across the mitochondrial membrane.

## Introduction

Many microorganisms (*Bacteria*, *Archaea*, single-celled eukaryotes) evolved versatile metabolic pathways for generating energy and for sustaining life under stressful environmental conditions. While significant progress has been made in identifying these metabolic pathways in bacteria and archaea, the mechanisms by which protists (single-celled eukaryotes excluding microbial fungi and algae) survive in environments where oxygen is low or absent and where high concentrations of sulfide and sulfidic compounds that suppress canonical aerobic respiration remain relatively unknown. The metabolic collaboration between eukaryotes and their symbiotic partners, including bacteria or archaea, as well as the acquisition of organelles from other eukaryotes, such as chloroplasts from algal cells—a process known as kleptoplasty—is now widely acknowledged across nearly all lineages of protists [1, 2]. In kleptoplasty, a heterotrophic protist or metazoan sequesters and retains algal chloroplasts for several weeks to months [3] or, perhaps, longer [4]. Kleptoplasty has been reported in certain ciliated protists (e.g. [5]) and foraminifera (e.g., [6–8]), typically from shallow waters, and the retained plastids are shown remain active, capable of performing light-dependent carbon fixation and, potentially, ammonium assimilation (e.g., [8]).

In California’s Santa Barbara Basin (SBB) at water depths far below the base of the euphotic zone, certain benthic foraminiferal species dominate the microeukaryotic meiofauna within the anoxic, highly sulfidic sediments [9], an environment sometimes considered an Ocean Worlds analog. A common feature in these foraminiferal species is that most host symbionts—including microbial partners or organelles such as sequestered chloroplasts—that may bolster independence from oxygen and provide the ability to detoxify or, perhaps, use hydrogen-sulfide dependent metabolisms. Understanding the metabolic innovations of the host and its associated organelles or symbionts in such environments provides insights into microbial eukaryote evolution on early Earth and, possibly into lifeforms awaiting discovery on other planetary bodies. The foraminifer *Nonionella stella* presents a fascinating case study because it harbors intact chloroplasts (Supplemental Fig. 1A–C) and is highly abundant in anoxic, highly sulfidic marine sediments far below the euphotic zone (i.e. >500 m, [4, 6, 9]). Further, there are copious peroxisomes in *N. stella* (Supplemental Fig. 1B and D) that work in concert with mitochondria to perform additional previously described metabolic activities [10]. The full extent of the physiological capabilities of the acquired chloroplasts is currently unknown. Previous gene expression analysis confirmed the diatom origin of *N. stella* chloroplasts as well as the high expression of Rubisco genes (*rbcL*/S) that encode a key enzyme in the Calvin–Benson–Bassham (CBB) cycle, suggesting potential chloroplast roles in carbon assimilation [11]. However, because those specimens were exposed to light prior to and during preservation, their expression of light-regulated genes may be a methodological artifact. Thus, we obtained metatranscriptomes from in situ-preserved samples unexposed to sunlight to assess the functionality of active metabolic pathways of *N. stella* in their natural deep-water sulfidic environment. This approach allowed us to also examine whether kleptoplasty confers the capacity to assimilate inorganic carbon and the role(s) of other metabolisms potentially advantageous to the host. Metatranscriptome analyses were combined with isotope-incubation experiments to investigate if *N. stella’s* metabolism supplies energy and reducing equivalents (i.e. ATP, NADH, NADPH) to the chloroplast for dark carbon fixation via the CBB cycle in the absence of dissolved oxygen.

## Materials and methods

### Sample collections

Samples for this study were collected from the SBB (Southern California, USA) on two research cruises: NA127 aboard E/V *Nautilus* in July 2021 and SP2213 aboard R/V *Robert Gordon Sproul* in July 2022. Aboard the E/V *Nautilus*, samples were obtained via the Remotely Operated Vehicle (ROV) *Hercules*. On selected ROV dives, measurements of incident sunlight were measured at depth. A QSP-2150 photosynthetically active radiation (PAR) sensor (Biospherical Instruments Inc., San Diego, CA, USA) was mounted to *Hercules*’ brow and connected to its electronic data logging system during four dives to the SBB seafloor (H1850, H1852, H1855, and H1856). These dives spanned daylight as well as nighttime hours. Before the dives, all *Hercules* and clump weight-/tether-system Medea lights other than those used for viewing and imaging were covered with opaque tape. When *Hercules* was immobilized on the seafloor, all uncovered light sources were extinguished for 10 minutes. During this time, the PAR measured incident light levels (logged in volts). The PAR sensor was calibrated, the dark offset value (reading in complete darkness) calculated, and this value subtracted from all field measurements.

An Aanderaa Oxygen Optode 3830 sensor mounted on *Hercules* was used to measure dissolved oxygen concentrations in surrounding (bottom) seawater. The sensor was calibrated approximately 9 months prior to use. Imagery of the seafloor surface and sampling were recorded with *Hercules*’ standard cameras and video capabilities.

For this contribution, we targeted sediments that supported a white *Beggiatoa*-like microbial mat, known to exist in SBB for many decades (e.g. [12–14]), indicating these sediments coincide with or were near the oxycline. Using *Hercules*’ manipulator arm, July 2021 sediment samples were collected in 6.2-cm and 8.2-cm inner-diameter pushcores. Once a core was successfully obtained (i.e. clear overlying waters, relatively flat and undisturbed sediment surface), it was quickly secured in a quiver holder mounted to *Hercules*’ front basket (Supplemental Fig. 2A and B). Three types of pushcores were obtained for this study: (i) “regular” pushcores to provide samples of living specimens for varied tasks; (ii) “large injector” pushcores for in situ preservation of foraminiferal populations; and (iii) “small injector” pushcores where in situ incubations were executed. As soon as possible after the ROV was recovered on deck, the cores were catalogued and subsampled as necessary. For live materials to be taken to our lab at WHOI, we sectioned the top ~2-cm of sediment into 100- to 250-ml HDPE bottles, filled to no header space with chilled bottom waters collected from Niskin bottles mounted on the ROV, and maintained at ~6°C until transport on blue ice to WHOI. Those samples provided specimens for MAG analyses. Metatranscriptomics, MAG, and sources of live specimen cores were collected in the vicinity of 34.29795, −120.06036 at 571-m water depth.

Large injector pushcores were similar to those used previously [15, 16]. The fixative reservoir of large injector pushcores was filled with RNAlater supplemented with red food coloring (Supplemental Fig. 2B) to provide better visibility during injections. Once the core tube was placed into the seafloor between reference lines, the ROV manipulator was used to squeeze the reservoir, which was connected to the corer head space via plastic tubing. Careful observation permitted visualization of the pink preservative. Sediments of these cores provided in situ-preserved *N. stella* populations that were used for metatranscriptome analyses. As soon as possible after core recovery, sediments were rinsed in 0.2 μm-filtered artificial seawater (FASW) and specimens isolated using a very fine sable brush. Cytoplasm-laden specimens were categorized into green (most common), pink, and white specimens (Supplemental Fig. 1C). Pools of ~25 *N. stella* of the same cytoplasmic color were carefully rinsed three times in FASW. Each pool was preserved (again) in 100 μl of DNA/RNA Shield (Zymo Research) and stored at 4°C. Once samples were taken to the shore-based lab, they were kept frozen at −80°C until RNA extraction. Due to in situ specimen abundance, time constraints and personnel limitations during the expedition, we isolated as many pools of each type of specimen as feasible within the available ship time. In total, we collected 8 pools for metatranscriptome analysis.

The reservoir of small injector pushcores (Supplemental Fig. 2C and D) was filled with ^13^C-labeled bicarbonate (H^13^CO_3_). Once these core tubes were placed in the seafloor, the bicarbonate was injected and allowed to incubate for about 23 hours until the ROV returned to collect them. These cores were processed with red lights once they were recovered on the ship with waters collected into exetainers preloaded with ZnCl_2_ and sediments collected into scintillation vials and amended with ZnCl_2_ (300 μl of a 0.5-M solution).

Aboard R/V *Robert Gordon Sproul* in July 2022, we initially profiled the dissolved oxygen concentrations along with salinity and temperature across the full water column via CTDO_2_-Niskin rosette cast, selecting a site near but sufficiently distant from a lander deployed on NA127. Our general sediment sampling area for this contribution was 34.29904–120.05631 at a water depth of 581 m. Dissolved oxygen was measured in the water column and within 3–5 m of the seafloor using two Seabird SBE43 sensors attached to the CTD-Niskin Rosette; these had been calibrated about 9 months prior to our use*.*

On this July 2022 cruise, sediment samples were collected using an MC800 multicore (Ocean Instruments; Supplemental Fig. 2E) or a Soutar boxcore [17]. After core recovery to the ship and confirmation of an undisturbed sediment–water interface with microbial mat, multicores were moved to a ~ 6°C cold lab and microsensors were used to obtain downcore profiles of oxygen, sulfide, and pH. After profiling, the overlying water was siphoned off and the upper ~2 cm of sediments were transferred to 100–250 ml HDPE bottles with the remaining volume filled with site water. Upon confirmation of an intact sediment/water interface in a Soutar boxcore, overlying water was siphoned off and the upper ~2 cm of surface sediments were transferred to 100–250 ml HDPE bottles and the remaining volume filled with site bottom water. Collected sediments were stored cold and dark until further processing. The varied sediment sample types and their analyses are presented in flow-chart form (Supplemental Fig. 3).

### Carbon uptake experiments

Upon return to the laboratory in 2022, cytoplasm-containing *N. stella* foraminifera specimens were hand-picked under room light or under red light (i.e. dark treatment) and placed into 12-ml exetainers (Labco, 757 W, United Kingdom), 10 foraminifera per exetainer. The exetainers were filled without headspace with filtered seawater amended with ^15^NO_3_^−^ (100 μM addition, 75–99 atom% final values) or ^15^NH_4_^+^ (50 μM, 99 atom% final values), H^13^CO_3_ (2.3 mM, 50 atom% final values) and sulfide (2 μM). All isotope salts were purchased from Cambridge Isotope Laboratory. The sealed vials were mixed by inversion and incubated in the dark at 6°C. Aliquots of the amended seawater (water samples) were taken to determine initial isotopic values. Additionally, a “No Amendment” treatment was performed that contained the addition sulfide and ^13^C-labeled carbon but no nitrogen amendment (i.e. no NO_3_^−^ or NH_4_^+^ addition). These “No Amendment” samples serve as a technical control to provide a frame of reference for interpreting incubation results. Finally, there were No Isotope Controls that served to measure the background carbon isotopic values. All treatments and controls were run in triplicate. Samples were destructively sampled at 4-hour and 20-hour by fixing with 300 μl of ZnCl_2_ (50 wt%/v) and mixing by inversion.

Additionally, on the July 2021 cruise, the reservoir of small injector pushcores (Supplemental Fig. 2C and D) was filled with filtered artificial seawater amended with ^13^C-labeled bicarbonate (~5 mM at 50 atom% H^13^CO_3_ final) and ^15^N-labeled nitrate (100 μM, 99 atom% ^15^NO_3_^−^). Once these pushcore tubes were placed in the seafloor with about 100 ml of headspace overlying the sediment/water interface, bicarbonate was injected and allowed to incubate for about 23 hours until the ROV returned to collect them. These pushcores were recovered near dawn/dusk and processed with red lights once recovered to limit surface light exposure. The top 2-cm of the cores was harvested, and the sediment split with ~1/2 partitioned into a scintillation vial and treated with 300 μl of a saturated ZnCl_2_ solution for preservation. Upon return to the shore-based laboratory, these sediments were sieved using a 63-μm mesh screen and the retained fraction partitioned into tin capsules for POC determination as described. These fractions were dried (60°C overnight), acidified with 300 μl of 12 N HCl overnight to remove inorganic carbon and dried again at 60°C (overnight). Additionally, at sea, the overlying waters of the pushcores were aliquoted into 12-ml exetainers preloaded with 300 μl of saturated ZnCl_2_ for later analysis. Samples were stored cold (5°C) until analysis. To determine N_2_ production, about 4 ml of the preserved exetainer water sample was analyzed using a Membrane Inlet Mass Spectrometer (MIMS) equipped with a liquid nitrogen cold trap (trapping CO_2_) and a 600°C, Cu-reduction column to remove O_2_. ^28,29,30^N_2_ was quantified using a quadrupole mass spectrometer (RGA100, Stanford Research Systems). Standards were made using temperature-controlled (6°C) saline solutions (3.5%, 3%, 2.5% and 0% NaCl) in equilibrium with the atmosphere. The remaining volume of the exetainer was acidified with 1 ml of phosphoric acid (85%) to convert dissolved inorganic carbon to carbon dioxide (CO_2_). CO_2_ was purged from the exetainer and analyzed at UCDavis using the protocols described.

### Determination of ^13^C uptake

Water and foraminifera samples were measured for ^13^C content at the UC Davis Stable Isotope Facility (SIF). To account for label atom percent, water samples from the exetainers were injected into a 12-ml, helium-purged exetainer preloaded with 1 ml of concentrated phosphoric acid to convert dissolved inorganic carbon to carbon dioxide (CO_2_). CO_2_ was then purged from the vial and measured by isotope ratio mass spectroscopy (IRMS) using the SIF’s standard protocols. Standard lithium carbonate samples run every 10 samples were used to correct for instrument drift. *N. stella* were filtered onto a glass fiber filter, dried overnight (60°C) and then packed into tin capsules. Foraminifera were acidified by adding 300 μl of a 12 N HCl solution to the tin capsules to remove inorganic carbon (DIC and foram test) and incubated overnight, and finally dried at 60°C (overnight). Control samples (all amendments but the ^13^C-label, No Isotope Control) were prepared in the same manner. ^13^C of the foraminiferal biomass was measured using an Elemental analyzer (Elementar Vario EL Cube) interfaced with a 20–20 IRMS at the SIF using standard protocols. Instrumental drift was corrected for using 4 or more reference samples per SIF protocol. Values are reported as δ^13^C vs Vienna PeeDee Belemnite (V-PDB).


$$ \delta{{}{}^{13}C}_{foram}=\left(\frac{{\left(\frac{{}{}^{13}C}{{}{}^{12}C}\right)}_{foram}}{{\left(\frac{{}{}^{13}C}{{}{}^{12}C}\right)}_{VPDB}}-1\right)\ast 1000 $$

To resolve the addition of labeled ^13^C to the *N. stella* biomass under the incubation conditions the excess ^13^C was calculated as Δ^13^C for the treatment *N. stella* over the control (unlabeled) conspecifics:


$$ \Delta{}{}^{13}C=\left(\ \delta{{}{}^{13}C}_{foram}-\delta{{}{}^{13}C}_{treatment\ control}\right) $$

### Estimations of C-uptake

Using an approximation of foraminiferal biomass and the determined isotopic values, a mass of C taken up and the percentage of total biomass can be calculated. To estimate the biomass of *N. stella (*we were unable to find literature values) we have scaled from published measurements of another foraminifera*, Ammonia beccarii*, to *N. stella* using the ratio of each taxon’s biovolume. Published values for the biomass and diameter of *A. beccarii* are 2.235 μg C/individual (average) and 1000 μm respectively [18]. *N. stella* is a much smaller organism with a diameter on the major axis of only ~300 μm. Approximating each organism as an oblate spheroid with a minor axis half that of the major axis we calculate the biovolume of each species by:


$$ {V}_{species}=\frac{4}{3}\pi{\left({axis}_{major}\right)}^2\left({axis}_{minor}\right) $$

The ratio of the determined biovolumes (0.027 *N. stella*:*A. beccarii*) can be used to scale the reported carbon mass of *A. beccarii* to that of *N. stella* (0.060 μg C/individual). With this assumption and assuming the change in the delta values from the uptake of ^13^C from the H^13^CO_3_^−^ pool (and not by respiration of ^12^C from a stored organic pool, which would also result in a minor enrichment), the mass of carbon added to the biomass pool can be calculated by:


$$ \left[{C}_{biomass}\right]\delta{{}{}^{13}C}_{measured}=\left[{C}_{biomass}\right]\delta{{}{}^{13}C}_{unamended}+\left[{C}_{add}\right]\delta{{}{}^{13}C}_{H13 CO3} $$

### Ribonucleic acid extraction and library preparation

All cell sorting for RNA isolation was conducted on ship in July 2021; sorted *N. stella* were preserved in DNA/RNA Shield from ZymoResearch (USA). RNA was isolated from each pool (sample) using Quick-RNA Microprep (Zymo Research). Total RNA was quantitated with a Qubit Fluorometer, RNA quality was assessed by Nanodrop, and RNA integrity was determined by Bioanalyzer (Agilent 2100) and TapeStation (Agilent 2200). A total of 8 samples, based of the color of their cytoplasm were assigned into three designations: 5 green-colored, 2 pink-colored and one white/colorless (Supplemental Fig. 1C). Extracts of all samples passed quality control and were used for RNA library preparation.

We prepared total RNA libraries using the Trio RNA-Seq Kit (Tecan, USA). The cDNA was generated from total RNA (host + bacteria + chloroplasts) using random and oligo (dT) primers. Libraries were then amplified using single-primer isothermal amplification (SPIA) and prepared with double-stranded cDNA fragmentation end repair to generate blunt ends, and adaptor ligation. To deplete the ribosomal RNA reads, we designed *N. stella*-specific ribosomal depletion probes. Libraries were then amplified and quantified by Bioanalyzer and TapeStation. Each sample was prepared using unique eight-base barcodes and sequenced on one flow cell of NovaSeq S4 (Illumina), achieving approximately 150 million reads per sample.

### Data processing of ribonucleic acid reads

Reads were quality checked using TrimGalore (https://github.com/FelixKrueger/TrimGalore), which uses the adaptor trimming tools Cutadapt (https://cutadapt.readthedocs.io/en/stable/) and FastQC (www.bioinformatics.babraham.ac.uk/projects/fastqc/). Quality-controlled fastq files of each of the eight samples were concatenated into one fastq file per library preparation to perform co-assembly of eight samples. De novo transcriptome assembly was performed using Trinity v2.8.4 to generate contiguous transcript sequences (contigs) [19]. Transcript abundance per sample was quantified with Salmon 0.12.0 [20]. The abundance profiles from short-read multimapping were then used in RapClust [21] to identify and group contigs representing different fragments of the same gene. The small sample size limited our ability to conduct statistical analyses among the three types, as we only had two pink samples and one white sample. This is insufficient for drawing reliable conclusions about variability between the sample types. Transcripts were then taxonomically and functionally annotated by a number of annotation tools. Coding regions were identified in the contigs with TransDecoder [19] and extracted for annotation. InterProScan, a wrapper tool that uses multiple annotation systems, was used on the nucleotide sequences to predict Pfam and TIGRFAM annotations. Coding regions were also translated to amino acid sequences with the EMBOSS tool Transeq and subsequently annotated with EggNOG-mapper v2 to provide IDs for KEGG (Kyoto Encyclopedia of Genes and Genomes) pathway and module and NCBI Cluster of Orthologous Groups. We retained all annotated reads in our data; relatedness to chloroplast, bacterial or eukaryotic genes was identified by top hits using the NCBI Basic Local Alignment Search Tool (BLAST) search against NCBI’s nonredundant protein database (Supplemental Table 1). Conserved protein domains were identified by applying the Subfamily Protein Architecture Labeling Engine (SPARCLE) on the NCBI conserved domain database.

### Short reads from ribonucleic acid and deoxyribonucleic acid mapping to diatom’s chloroplast genomes

**Figure 1:**
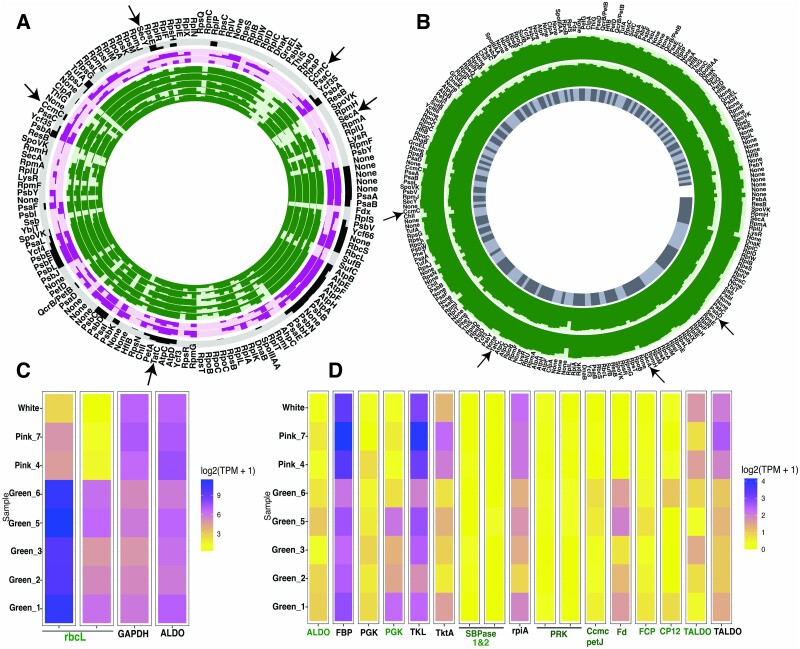
(A) Expression of sequestered chloroplast genes from SBB *N. stella* metatranscriptome reads. RNA-reads of *N. stella* mapped onto the chloroplast genome of the diatom *Skeletonema pseudocostatum.* Rings in green represent the green-colored *N. stella* samples (*n* = 5), rings in pink represent the pink-colored *N. stella* (*n* = 2) samples, black ring represents the white-colored *N. stella* sample. Ring height is maximum detection of each gene. The outermost ring displays gene category (COG) for all chloroplast genes. None: refers to unannotated genes. Ccmc, TatC, SecA, and Y genes are labeled by black arrow. (B) *S. pseudocostatum* chloroplast genome, assembled from metagenomic-reads obtained from two samples of green *N. stella*. Gene-level coverage was log10-transformed; outermost ring displays gene category (COG) for all chloroplast genes. Inner grey ring represents the 113 contigs with 221 genes assembled from the DNA short reads. (C and D) Expression of transcripts of CBB cycle and related genes. Rubisco subunit (*rbcL*); GAPDH (glyceraldehyde-3-phosphate dehydrogenase); fructose-bisphosphate aldolase (ALDO); fructose-bisphosphatase (FBP); phosphoglycerate kinase (PGK); transketolase (TKL); thiamine diphosphate (TKtA); sedoheptulose-bisphosphatase (SBPase); ribose 5-phosphate isomerase A (rpiA); phosphoribulokinase (PRK); cytochrome C6 (PetJ/Ccmc); ferredoxin (Fd); fucoxanthin chlorophyll a/b protein complex (FCP); chloroplast protein (CP12); transaldolase (TALDO). Sequences classified as diatom have green labels.

*N. stella* RNA and DNA short reads were obtained from the three types of *N. stella* holobionts (green, pink, and white) based on the color of the cytoplasm, where green represents the type that harbors chloroplasts, while pink and white harbor few to no chloroplasts, respectively, as evidenced by plastid gene expression analyses (Fig. 1A). Both DNA and RNA short-reads were mapped with bowtie2 onto reference plastid genomes downloaded from NCBI GenBank for *S. pseudocostatum* (GenBank accession no. MK372941). The read mapping produced coverage values for each reference genome by counting the number of times that a nucleotide was included in a short-read alignment to the reference. The gene-level coverage was defined as the average nucleotide’s coverage in that gene, and a nonzero coverage value indicates that the gene was detected in the transcriptome. The coverage and detection information were visualized with anvi’o [22]. In both DNA and RNA-short reads, the *S. pseudocostatum* plastid had the deepest and broadest coverage with the fewest mismatches (SNPs). Gene-level coverage was log10-transformed for display.

### Identification of key proteins

We searched our host and holobiont transcriptomic databases for encoded amino acid sequences involved in CBB, mitochondrial oxidative phosphorylation, sulfur oxidation and reduction, multicopper oxidases, and genes associated with pyrophosphate metabolism. Reference sequences were obtained from NCBI RefSeq nonredundant proteins database, EggNOG, Uniprot, Laccase and Multicopper Oxidase Engineering Database (https://lcced.biocatnet.de), and published genome and transcriptome of *Globobulimina* sp. [23]. Genes were identified on the basis of annotations using the EggNOG database. Amino acid sequences were clustered at 95% identity using CD-HIT (Cluster Database at High Identity with Tolerance) for the identification of representative sequences in each cluster. Amino acid analysis showed that H^+^- PPase of *N. stella* exhibited the key conserved motifs underlined in the following sequence: GGGIYTKAADVGADLVGKVESAIPEDSPKNPATIADNVGDNVGD [24, 25].

### Organelle localization predictions

Chloroplast and mitochondria localizations were predicted using PredSL and TargetP version 2.0 [26, 27] (Supplemental Table 1). Localization predictions of PredSL is based on homology using Markov Chains and hidden Markov model for the prediction of the subcellular localization of proteins in eukaryotic cells from the N-terminal amino acid sequence. Whereas localization predication using TargetP sorts the proteins signals at the N-terminal with targeting peptides to mitochondria or chloroplast. Confidence levels were assigned to the putative mitochondrial proteins by integrating the combined probability of localization and the homology identifications to the reference mitochondrial protein database. Both methods have been applied previously [10] for two benthic foraminiferal species, *N. stella* and *Bolivina argentea*. Proteins that were not localized to mitochondrion or chloroplast were classified as cytosolic and were included in the metabolic reconstruction. Taxonomic assignment was based on DIAMOND BLASTP and NCBI BLASTP, all proteins presented in this study were annotated by EggNOG and KEGG and had at least 50% coverage to a reference protein from NCBI and DIAMOND BlastP database [28].

### Cell sorting, deoxyribonucleic acid extraction, and library preparation

Sediments preserved in situ with RNAlater was processed in the lab following our established protocol [11]. DNA from green *N. stella* cells was extracted from two pools of cells, one had 715 cells, while the second had 110 cells. DNA was extracted using the Quick-DNA Microprep kit (Zymo Research, USA). Total DNA was quantitated with Nanodrop and Tapestation (Agilent 2200) and the total DNA concentration for the first sample was 240 ng and for the second was 89 ng. Libraries were prepared using Nextera XT DNA library preparation kit (Illumina, USA) and sequenced on NovaSeq S4 (Illumina).

### Metagenomics assembly

Metagenomic reads were quality checked using TrimGalore (https://github.com/FelixKrueger/TrimGalore), which uses the adaptor trimming tools Cutadapt (https://cutadapt.readthedocs.io/en/stable/) and FastQC (www.bioinformatics.babraham.ac.uk/projects/fastqc/). Reads were downsampled using bbnorm version 38.90 with a target coverage of 100x. Downsampled reads were assembled using spades version 3.15.1 in the metagenome assembly mode [29]. Assembly metrics were calculated with quast version 5.0.2 [30]. Gene calling was performed using genemark_ES with a minimum contig length of 5000 [31]. Resulting gene calls were annotated using eggnog mapper version 2.1.7 [32]. Reads were mapped to the assembly using bbmap version 38.90, sorted and indexed with samtools version 1.9 [33], and counted using the pileup.sh script from bbmap. Finally, bins were assembled using metabat version 2.15 [34]. We acknowledge that assembling the *N. stella* genome from metagenomic reads presented significant challenges due to the lack of effective assemblers for large and repetitive genomes. Consequently, the results presented in our manuscript are based on assembled contigs rather than a complete genome assembly.

## Results

### Environmental conditions

A conspicuous and extensive white microbial mat of sulfur-oxidizing bacteria has been documented on the seafloor in the deeper parts of SBB for decades (e.g. [12–14, 35]). This mat was well developed and extensive in both July 2021 (Supplemental Fig. 2A–D) and July 2022 (Supplemental Fig. 2E). It was present during four ROV *Hercules* dives (H1850–1852, H1855), to sites separated by as much as 4.2 km, in July 2021, between water depths of 569–580 m, and it appeared on the surface of each of 6 boxcores taken in July 2022 between water depths of 579 to 584 m, the deepest water-depths sampled at that time. In the deepest areas, the microbial mat varied in surface appearance, where thickly clumped spots transitioned into finely tufted areas (Supplemental Fig. 4A and B). When sampled using a pushcore or scoop, the concentrated surface distribution was clearly discernible (Supplemental Fig. 2B inset and Supplemental Fig. 4C). As described previously (e.g. [13]), the sediments beneath the surface of the microbial mat are finely laminated. Cohesive microbial mats were not observed at shallower sites in either 2021 (462–470 m) or 2022 (554–556 m), however, some filaments of sulfur-oxidizing bacteria were noted during microscopic examination of those sediments.

In the Basin’s deeper regions, dissolved oxygen was undetectable in bottom waters in July 2021, and minimal in July 2022 at ~0.4 μmol/l in bottom waters ~3–5 m above the seafloor. Data from the PAR sensor indicated that sunlight was not detectable on the seafloor during any of the 2021 dives; PAR was not measured during the 2022 cruise.

### *Nonionella stella* characteristics

The typical color of cytoplasm-laden *N. stella* (i.e. living specimens) is dark brownish green (Supplemental Fig. 1C; see also [11]); however, we also found considerable populations of pink and white cytoplasm-laden *N. stella* in July 2021 (Supplemental Fig. 1C). The test (shell) morphologies of all three populations appeared identical, so we assumed all three *N. stella* color variations were conspecifics. This was confirmed using 18S rRNA analysis of assembled metatranscriptome contigs. Although enumeration of *N. stella* abundance was not a focus of this study, the population in July 2021 was particularly abundant, approaching densities observed in October 1996 when over 230 *N. stella* cm^−3^ were recovered [36]. *N. stella* abundances in July 2022 were also high, but not quantified.

### Transcriptomic analyses supporting autotrophic C fixation

RNA and DNA short sequence reads of the *N. stella* samples were mapped to the chloroplast genome from the diatom *Skeletonema pseudocostatum*, a reference genome retrieved from NCBI, which emerged as the closest relative to the sequestered plastids, consistent with our previous findings [11]. The assembled plastid genome is comprised of 113 contigs with 221 genes. Kleptoplast genes, *rbcL/S*, and almost all subunits of light energy reaction centers PSI and PSII, as well as ATP synthase (ATPase) were all highly expressed in the metatranscriptome data (Fig. 1A, C). Moreover, the subunits of cytochrome b_6_f complex, a key component in the photosynthetic electron transfer process, were expressed in our metatranscriptome data and were encoded in the assembled chloroplast genome derived from both DNA and RNA sequence reads from green *N. stella* pools (Fig. 1A and B).

The *S. pseudocostatum* reference genome does not encode plastocyanin, a known photosynthetic electron carrier that mediates electron transfer from PSII to PSI via the cytochrome b_6_f complex [37]. The expression of a nuclear-encoded plastid-targeted *petJ* gene coding for cytochrome C6 was detected in the green *N. stella* samples (Fig. 1D). Cytochrome C6 can be an alternative electron carrier between cytochrome *b*_6_*f* complex and photosystem I for diatoms and can replace plastocyanin [38–40]. Findings included the expression of the gene for the plastid-encoded cytochrome C-type biogenesis protein (CcsA/CcmC), a key protein of cytochrome C maturation, and the presence of this gene in the chloroplast genome from assembled DNA reads of the green *N. stella* samples, but not the pink and white *N. stella* samples (Fig. 1A, B, and D).

The fucoxanthin chlorophyll a/b protein complex (FCP), a nuclear-encoded and plastid-targeted gene, was detected in the metatranscriptome of the green *N. stella* samples although its relative expression was lower than in the previously reported data [11] (Fig. 1D). This lower expression suggests that although the *fcb* gene was retained by *N. stella*, potentially within its nuclear genome, its expression may be influenced by exposure to light. The gene for another diatom nuclear-encoded and plastid-targeted protein (CP12) was solely detected in the green *N. stella* samples, in both metatranscriptome and MAG data. A recent study showed that CP12 in the diatom *Thalassiosira pseudonana* is constitutively expressed under both light and dark conditions and forms a complex with Ferredoxin-NADP reductase (FNR) and Glyceraldehyde 3-phosphate dehydrogenase (GAPDH; GAPDH-CP12-FNR) [41]. Ferredoxin (Fd), an iron–sulfur protein that mediates electron transfer in diatoms, was also expressed in our dataset (Fig. 1D).

The metatranscriptome data revealed high expression of the central CBB gene, *rbcL* (Fig. 1C), which constitutes the largest fraction of non-foraminifera eukaryotic transcripts in our dataset. Other CBB-associated genes, including phosphoribulokinase (PRK) and sedoheptulose-1,7-bisphosphatase (SBPase), both of which are nuclear-encoded plastid-targeting proteins, exhibited distinct results. While PRK was detected in the MAG data, it had very low expression values in the metatranscriptomes, whereas SBPase expression was not detected (Fig. 1D).

**Figure 2:**
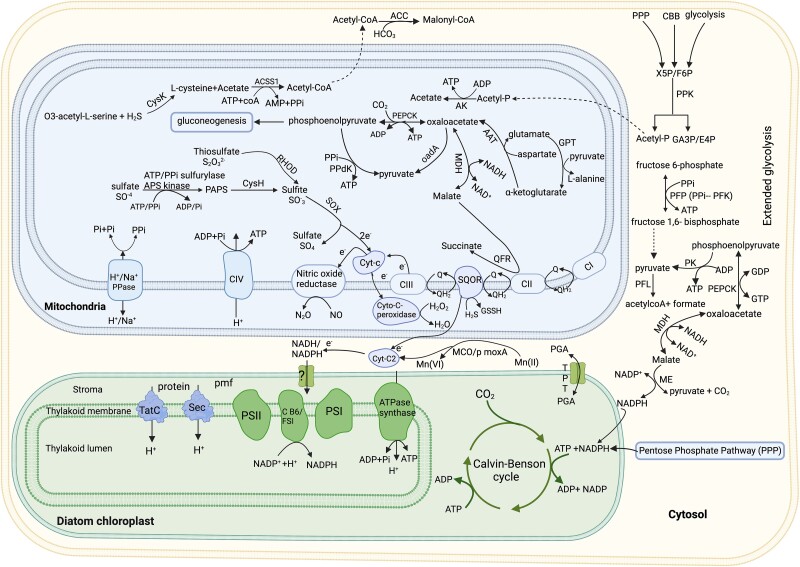
Key metabolic pathways in *N. stella* and their kleptoplast. Schematic representation of metabolic pathways in the foraminifera *N. Stella* based on gene expression data from the metatranscriptome reads obtained from in situ preserved samples. Identified proteins and metabolic pathways in carbon metabolism: phosphoenolpyruvate carboxykinase (PEPCK); pyruvate kinase (PK); pyruvate-phosphate dikinase (PPi-dependent PPdK); oxaloacetate decarboxylase (oadA); malate dehydrogenase (MDH); aspartate aminotransferase (AAT); glutamate pyruvate transaminase (GPT); acetyl-CoA carboxylas (ACC); phosphoketolase (PPK); acetyl-CoA synthetase (ACSS1); malic enzyme (ME); acetate kinase (AK); and pyruvate formate lyase (PFL). Sulfur cycle, including sulfate assimilation proteins: ATP/PPi sulfurylase-APS kinase and CysH or phosphoadenosine 5′-phosphosulfate reductase (PAPS). Sulfite and thiosulfate oxidation proteins: sulfite oxidase (SOX) and rhodanese (RHOD). Electron-transport chain; NADH: ubiquinone oxidoreductase (complex I); succinate dehydrogenase (complex II); ubiquinol–cytochrome c oxidoreductase (complex III, or cytochrome bc_1_ complex); nitric oxide reductase (complex IV) and ATP synthase (complex V); c-type cytochromes; (Cyt-C) cytochrome c2 (Cyt-C2); cytochrome-C peroxidase (Cyto-C-peroxidase); H^+^ proton-pumping pyrophosphatase (PPi synthase); and manganese oxidase (moxA). Kleptoplasty proteins and associated metabolism: photosystem I (PSI); photosystem II (PSII); cytochrome b6f complex (Cb6/f complex); secretory pathway (Sec); twin-arginine translocation pathway (TatC); triose phosphate transporter (TPT); and 3-phosphoglycerate (PGA).

Transcripts identified as diatom Tat and Sec pathways (i.e. twin arginine transporter (Tat)-dependent and secretory (Sec)-dependent import pathways; Fig. 1A and B) were consistently expressed in all green *N. stella* samples. These pathways are vital for transporting proteins across the thylakoid membrane into the lumen [42]. The expression of chloroplast ATP synthase subunits, the cytochrome b6f complex subunits, and photosystem I and II indicates the functionality of the chloroplast (Figs 1A and B, and 2). The expression of protein translocating pathways (Tat and Sec) suggests that the proton motive force (pmf) is active, possibly to deliver exogenous reducing equivalents and energy from stroma to lumen for carbon fixation by CBB [42]. However, the expression data alone cannot confirm the activity in maintaining the redox potential. We must note it remains uncertain whether ATP synthase is expressed exclusively for ATP synthesis or if it also has a role in ATP hydrolysis to uphold chloroplast lumen homeostasis.

### Isotope incubations supporting autotrophy

In July 2021 (NA127), all push cores used for seafloor incubations (with ^13^C-labeled bicarbonate, *n* = 3) were obtained from the same site (571 m); the core liners deployed with minimal disruption of the sediment/water interface and incubated over the same time period. After placement of the core liners, H^13^CO_3_^−^ was injected into the sealed headspace of the core liner by squeezing the label into the cores’ overlying waters from a remote reservoir and without removing the core from the seafloor. After 23 hours, the cores were preserved in situ with ZnCl_2_. Net production of N_2_ in the cores (sediments + porewaters) averaged 1.84 ± 1.48 nmol N_2_/cm^2^/day. Net N_2_O production was lower, at 13 ± 6.5 pmol/cm^2^/day. δ^13^C values of the POC from the push cores were near values expected for marine particulate organic matter (POC) and similar to incubations of unlabeled, pooled *N. stella* (Supplemental Fig. 5). Of note, this POC measurement was on material retained on a 63-μm sieve. δ^13^C values of the POC ranged from −20.6‰ to −22.7‰, with a mean of −21.9 ± 0.7‰.

The δ^13^C values for isolated pools of green *N. stella* from dark incubation experiments performed in July 2022 (SP2213) showed uptake (i.e. shift in δ^13^C value) of H^13^CO_3_ into biomass in all treatments relative to the unlabeled controls (Table 1; Supplemental Fig. 6A and B). Incubations amended with ammonium displayed the greatest uptake of H^13^CO_3−_ into biomass, with up to a ~ 3‰ shift in delta-values over the 20-hour incubation for *N. stella* isolated under laboratory / sunlight conditions as well as *N. stella* isolated under minimal (red) light. Similar trends, with smaller amplitude change (<+1 per mil), were observed in 20-hour nitrate-amended incubations.

**Table 1:** Shifts in isotopic values resulting from incubations with a ^13^C-labeled inorganic carbon source.

	*n*	δ^13^C-POC	90% confidence interval	Δ^13^C	90% confidence interval	% change in biomass carbon
Ammonium + ^13^C	5	−18.91	0.65	3.20	0.42	0.57
Nitrate + ^13^C	5	−21.52	0.35	0.93	0.34	0.13
^13^C	2	−22.11	0.43	0.34	0.20	0.09
No-Isotope Control	2	−22.45	0.31	0.00	0.31	0.00

### Mitochondrial respiratory electron transport chain and proton pumps

**Figure 3:**
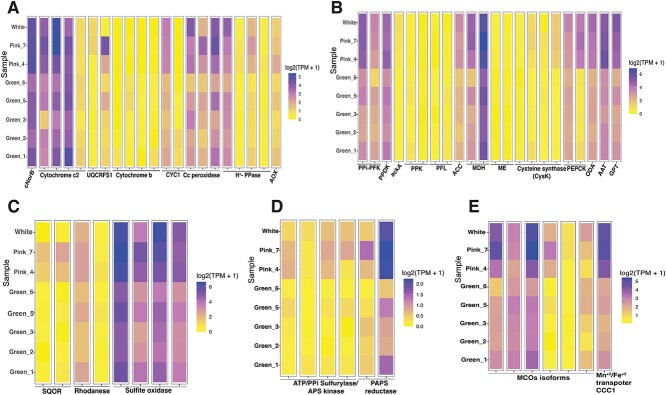
Heatmaps showing the log_2_- transformed TPM (transcript per million) of each cluster (columns) in the different *N. stella* samples presented by cytoplasmic color (rows) for all genes in (A) electron transport chain and cytochromes, proton pumps; (B) carbon metabolism; (C) sulfur oxidation; (D) sulfur reduction; (E) multicopper oxidases domain and iron/manganese transporter. Abbreviations align with those in Supplementary Table 1.

In agreement with our previous study [10], expression of genes for electron transport chain (ETC) complexes, complex I and II were detected in our data, as well as alternative oxidase (AOX; data in Supplemental Table 1). The expression of cytochrome b (*cob*), one of the three subunits of the respiratory cytochrome complex III, has 99% amino-acid sequence similarity to diatom *cob*, suggesting that *cob* in *N. stella* is of diatom origin (Fig. 3A). The expression of this *cob* was represented by only one isoform and detected only in the green samples, whereas very low to no *cob* expression was observed in the white and pink samples. Analysis based on mapping RNA short reads of the *N. stella* metatranscriptome into the *Skeletonema pseudocostatum* mitochondrial reference genome [37] revealed a fragmented mitochondrial genome, except for *cob* and *NADH dehydrogenase*, both of which maintained consistent expression across the green samples. This result suggests that not all mitochondrial genes for this diatom are actively transcribed in *N. stella* (Supplemental Fig. 7A), but rather only selected genes. Additionally, MAG analyses showed that the *cob* gene was encoded in a contig that comprises diatom NADH dehydrogenase subunit 5 at the 5′ region and *cob* at the 3′ region, with about 10 kb of intergenic region between them (Supplemental Fig. 7B). The two other subunits of complex III (ubiquinol-cytochrome c reductase, cytochrome C1) are both classified as foraminiferal/eukaryotic genes. The consistency of *cob* expression across the green samples suggests that the *N. stella* acquired diatom mitochondria. Inter-mitochondrial gene transfer has been documented between distantly related plant species [43]. *N. stella* may also have acquired *cob* horizontally from phagocytosed diatom mitochondria or through a viral vector. Horizontal gene transfer of mitochondrial genes has been reported among fungal species [44]. This acquisition of *cob* genes underscores the functionality of complex III in *N. stella*’s ETC (Fig. 3A), however, this functionality was limited to only the green samples.

In contrast to previous findings in experimentally manipulated *N. stella* [10], our current gene expression analysis indicates that aerobic respiration is likely not sustained in the *N. stella* samples that were preserved in situ. This conclusion is based on incomplete expression of respiratory complex IV (known as cytochrome C oxidase in the ETC), with expression observed only for certain subunits (Supplemental Fig. 8). Thus, the incomplete functionality of complex IV does not support aerobic respiration but may instead contribute to alternative functions. A similar observation was reported in protists with mitochondria-related organelles (MROs) including some anaerobic ciliates [45]. An additional finding in our current analysis is that nitric oxide reductase (cytochrome c; NorB; EC: 1.7.2.5) that catalyzes the reduction of NO to N_2_O, was highly expressed in all samples (Fig. 3A). This is in agreement with our previous findings for incubation experiments in *N. stella* under different oxygen regimes [11]. Our current metatranscriptome data detected three isoforms of *cnorB*, with one localized to mitochondria, suggesting its role in supporting denitrification and electron transfer from complex III through cytochrome C1 to nitric oxide as the terminal electron acceptor. Sequence analysis of transcripts revealed that one of the isoforms of *cnorB* was encoded in the same transcript with mitochondrial eukaryotic Cullin protein. A BLASTp sequence search and conserved domain analyses showed a striking similarity between Cullin from our *N. stella* transcripts and Cullin from *Reticulomyxa filosa*, a freshwater foraminifera. This finding strongly suggests that *cnorB* is likely encoded in the foraminifera genome.

Additionally, *N. stella* expressed four proteins annotated as C-type cytochromes, all of which were highly expressed, and having the conserved motif CXXCH (Figs 2 and 3A). This highlights the importance of these cytochromes in *N. stella’*s ETC. Transcription of all ATPase subunits across all samples suggests that SBB *N. stella* likely possesses the ability to generate ATP by harnessing proton gradients created by respiratory complexes I, III, and NorB (Fig. 2). Alongside the expression of proton-pumping V-type ATPase, our dataset further unveils the expression of H^+^- translocating inorganic pyrophosphatase (H^+^- PPase), sometimes referred to as PPi synthase (Figs 2 and 3A). This electrogenic proton pump plays a critical role in scavenging inorganic pyrophosphate (PPi) within the cell’s cytoplasm and mitochondria, facilitating proton transport and energy generation from mitochondria to cytoplasm, potentially generating a proton gradient across the mitochondrial membrane. Alternatively, H^+^- PPase might use the proton gradient to synthesize PPi in mitochondria to be utilized as an energy source by some enzymatic pathways. In plants, it was shown that under stressful conditions where oxygen is low or absent, ATP generation slows or even stops [46]. In the absence of oxygen, H^+^- PPase generates PPi to replace ATP in certain glycolytic pathways that are pyrophosphate dependent, thus minimizing ATP use [46].

Cytochrome C peroxidase (Ccp), a common enzyme that protects against oxidative stress [47], was highly expressed across all *N. stella* samples and, based on protein sequence analysis, is localized to the mitochondria (Figs 2 and 3A; Supplemental Table 1). In bacteria, Ccp serves as a respiratory enzyme that donates electrons to H_2_O_2_ under anoxia [48]. In the mitochondria of aerobic yeast, Ccp and Cytochrome c form a complex with ferrocytochrome C in the inter-membrane cristae, where Ccp reduces hydrogen peroxide to water using electrons provided by ferrocytochrome C. This safeguards the cell against peroxide toxicity and maintains aerobic respiration [49]. On the basis of downregulation or absence of the expression of cytochrome c oxidases, aerobic respiration is unlikely to be active (Supplemental Fig. 8). This implies that Ccp may play a role in an unconventional pathway that supports H_2_O_2_ reduction (Fig. 2). We also detected expression of genes involved in menaquinone (MK) biosynthesis in all three colors of *N. stella* based on our metatranscriptome analysis. Menaquinone is an electron carrier for both aerobic and anaerobic processes known to reduce Ccp under anoxia [50].

### Heterotrophic organic carbon metabolism

*N. stella* expressed a suite of anaerobic carbon metabolisms, including pathways for acetate production and utilization, pyruvate metabolism, and the generation of precursors essential for anabolic pathways (Fig. 3B). These pathways, described in greater detail below, illustrate an adaptation of this eukaryote’s carbon metabolism to an anoxic, dark environment.

In all *N. stella* metatranscriptome samples (green, white, and pink), we detected the expression of the gene for pyruvate formate lyase (PFL), an oxygen-sensitive enzyme that plays a role in anaerobic glucose fermentation in obligatory or facultative anaerobic bacteria and eukaryotes [51]. PFL expression was highest in the green *N. stella* samples (Fig. 3B). This enzyme operates and is activated through a glycyl radical-based homolytic mechanism, converting pyruvate into acetyl-CoA and formate [52, 53]. PFL has significant importance in fumarate-independent anaerobic glycerol metabolism [54].

Another metabolic pathway involved in anaerobic carbon metabolism was identified in *N. stella*: the phosphoketolase (PPK)-acetate kinase pathway. PPK uses xylulose 5-phosphate (X5P) or fructose 6-phosphate (F6P) generated by the oxidative branch of the pentose phosphate pathway (PPP) [10] as a carbon source to produce glyceraldehyde-3-P (GA3P) or erythrose-4-P (E4P) and acetyl phosphate (Acetyl-P) [55]. Amino acid-sequence analysis revealed that PPK expression was unique to the green *N. stella* samples (Fig. 3B), and the *N. stella* PPK protein was identical to the diatom PPK protein. Additional steps in this pathway involve ATP production and acetate production from acetyl-P catalyzed by the mitochondrial acetate kinase (Figs 2 and 3B). This pathway was identified in bacteria and a few anaerobic eukaryotes [56]. The acetate is ultimately converted to acetyl-CoA through the action of acetyl COA synthase (ACS). This finding illustrates *N. stella*’s unique strategy of adapting to anaerobic carbon metabolism, allowing it to thrive within a distinct ecological niche.

We detected pathways for acetate formation in all three types of SBB *N. stella*. Several isoforms of cysteine synthase (cysK) were expressed (Fig. 3B), an enzyme that uses hydrogen sulfide that exists in considerable concentrations in these pore waters [12, 57], and *O-*acetylserine, to generate L-cysteine and acetate. Localization analysis indicated that *cysK* isoforms have both mitochondrial and cytosolic localization. To date, the cysteine synthase pathway is only identified in plants, bacteria and ciliates [58, 59]. *N. stella*’s *cysK* has the three conserved lysine residues (Lys66, Lys77, Lys226) required for enzyme activity [58].

Based on in-depth gene expression analyses, a series of enzymes pivotal to oxaloacetate decarboxylation and pyruvate generation have been detected within SBB *N. stella*. Oxaloacetate decarboxylase (oadA), a mitochondrial enzyme responsible for the irreversible conversion of oxaloacetate into pyruvate, was consistently expressed in our *N. stella* samples along with genes for additional decarboxylation enzymes (Fig. 3B). Among them, phosphoenolpyruvate carboxykinase (PEPCK) was identified, which plays a role in the reversible transformation of phosphoenolpyruvate (PEP) into oxaloacetate [60, 61]. PEPCK was present in two isoforms: ATP- and GTP-utilizing PEPCKs, which were localized to the mitochondria and cytosol, respectively (Figs 2 and 3B). Another decarboxylation enzyme detected was malate dehydrogenase (oxaloacetate-decarboxylating NADP and/or NAD-malic enzyme), which generates pyruvate and NADPH from malate (Figs 2 and 3B). Because *N. stella* does not express pyruvate carboxylase [10], a mitochondrial enzyme responsible for oxaloacetate synthesis from pyruvate, we suggest that PEPCK is involved in pyruvate synthesis. We hypothesize that *N. stella* adapted glucogenic metabolism to generate carbohydrates using PEPCKs, which catalyze the generation of PEP from oxaloacetate. The glyoxylate cycle [10], as well as the expression of aspartate aminotransferase (AAT), can both contribute to oxaloacetate production. We also predict that produced glutamate from ammonium assimilation [11] is converted by Glutamate-pyruvate transaminase (GPT) to alpha-ketoglutarate, suggesting that both enzymes are involved in coordinating carbon and nitrogen metabolism (Figs 2 and 3B).

Our data demonstrate that *N. stella* uses enzymes that depend on inorganic pyrophosphate as an energy source for carbon metabolism. The expression of PPi-dependent enzymes, the cytosolic PPi-PFK and the mitochondrial phosphate pyruvate dikinase (PPi-PPdK), supports this hypothesis because these glycolysis enzymes can catalyze the generation of ATP. In contrast, we detected the expression of five malate dehydrogenase (MDH) isoforms: four were localized to the cytoplasm, while one was mitochondria-localized; at least two of these isoforms were previously reported [10]. MDH catalyzes the reversible reaction of malate into pyruvate and CO_2_ generating NADH or NAD^+^ depending on the directionality of the reaction. The NADH and NADPH generated through cytoplasmic malate dehydrogenase and malic enzyme can potentially be used as reducing powers for carbon fixation in the kleptoplast via the CBB (Figs 2 and 3B). However, further investigations are required to confirm this pathway functions in SBB *N. stella*.

We searched our transcriptome data for carboxylation enzymes that may be involved in anaplerotic CO_2_ fixation to replenish TCA-cycle intermediates, a process known as heterotrophic CO_2_ fixation via anaplerosis [62]. Enzymes that replenish the oxaloacetate pool (i.e. pyruvate carboxylase and phosphoenolpyruvate carboxylase [PEPC]) were not expressed. However, we detected expression of Acetyl-CoA carboxylase (ACC; Fig. 3B), an enzyme that catalyzes the irreversible carboxylation of Acetyl CoA to manoyl CoA, a key metabolite precursor for fatty acid synthesis [63], suggesting that anaplerosis in *N. stella* does not replenish TCA-cycle intermediates.

*N. stella* has evolved a variety of metabolic pathways that enhance its ability to produce reduced NADH and NADPH. Key pathways include mitochondrial and cytosolic malate dehydrogenases, which play significant roles in NADH/NADPH generation. Additionally, the oxidative PPP serves as a primary source of NADPH, whereas the malic enzyme also contributes to NADPH production. Additionally, [FeFe]-hydrogenase (HydA, which is fused with NuoG and the small subunit of iron hydrogenase) have been previously described [10, 11] as contributors to NADH and NADPH generation. The NADH and NADPH produced by these pathways, especially those occurring in the cytosol, may be transported to the kleptoplast for utilization in the CBB cycle. In our metatranscriptome analysis, we identified the triose-phosphate transporter family, which facilitates the exchange of metabolites between the cytosol and the chloroplast, and NAD(P) + transhydrogenase (NNT) E.C. 1.6.1.2 in our metatranscriptome dataset (Supplemental Table 1). These findings highlight the diverse metabolic capabilities of *N. stella*.

### Inorganic sulfur compound oxidation and reduction are key pathways in *Nonionella stella*

Our data indicate that all in situ-preserved *N. stella* (green, pink, and white) are capable of oxidizing hydrogen sulfide using sulfide quinone oxidoreductase (SQOR), indicating that the previously reported expression activity [10] is active in situ. *N. stella* also expresses at least two isoforms of Rhodanese or Rhodanese-like enzyme that was localized to mitochondria and belongs to the sulfurtransferase family of enzymes present in organisms from all three domains of life (Fig. 3C). Rhodanese catalyzes the transfer of a sulfur atom from thiosulfate (sulfur donor) to cyanide (sulfur acceptor), producing sulfite and thiocyanate. Rhodanese minimizes build-up of the toxic compound sulfane sulfur and acts as a supply of sulfur [64, 65]. Thus, rhodanese may play roles in channeling sulfur metabolism in *N. stella*. The produced sulfite may serve as a source of electrons in *N. stella*. We identified three sulfite oxidase isoforms, enzymes responsible for sulfite oxidation to sulfate (Fig. 3C), all highly expressed in our data. Only one of these isoforms has been reported in the literature [10, 23].

Assimilatory sulfate reduction genes were also detected in our data. Sulfate can be converted into sulfite using two different enzymes. The first enzyme is a bifunctional sulfate adenylyltransferase/adenylyl sulfate kinase forming APS as an intermediate and then PAPS (3’-Phosphoadenosine-5′-phosphosulfate) as a final product. PAPS is then reduced to sulfite with 3′-phosphoadenosine-5′-phosphosulfate reductase (PAPS reductase, commonly known as cysH-thioredoxin) [66]. Both enzymes have mitochondrial localization based on our prediction analysis (Supplemental Table 1, Fig. 3D). Our analysis cannot determine whether the sulfate reduction enzymes are ATP-dependent or PPi-dependent enzymes. Sulfite reductase, which catalyzes the conversion of sulfite to hydrogen sulfide, was not expressed in any sample, indicating that *N. stella* may perform incomplete sulfate reduction to sulfite. The expression levels of sulfate reduction genes are markedly lower compared to those of sulfite oxidation genes, with a ratio of 1:1000 (Fig. 3C and D). Such findings suggest that the *N. stella* is not allocating significant energy resources towards sulfate reduction. To understand its role, further experimental investigations are required. Collectively, these data demonstrate that both assimilatory sulfate reduction and sulfite oxidation pathways coexist in SBB *N. stella.*

*N. stella* expressed four multicopper oxidase (MCO) proteins (Fig. 3E) that range in size from 250 to 445 amino acids. Conserved domain-search revealed that *N. stella* MCOs all belong to the two-domain multicopper oxidases (2dMCO) that consist of 2 cupredoxin-like domains and copper ligands arranged in four conserved motifs (HXHG, HXH, HXXHXH, and HCHXXXHXXXXM/L/F). This is a typical structure for bacterial MCOs and bacterial and eukaryotic laccases [67, 68]. MCO enzymes are exported across outer membranes and can oxidize various metals such as Fe (II), Cu (I), and Mn (II). We did not detect the expression of manganese peroxidase in our data. Certain bacteria can oxidize Mn (II) as an electron source to supplement the energy required for chemolithoheterotrophy [69]. Localization analysis supports that *N. stella* MCO genes have signal peptides enabling the translocation of MCO proteins into membranes of organelles or extracellular vesicles. Only one of the MCO genes has a mitochondrial localization signal (Supplemental Table 1). In general, most copper-containing proteins are extracellular enzymes [68]. These *N. stella* MCOs were annotated as manganese oxidase (moxA; E.C. 1.16.3.3) based on KEGG annotation. Protein domain analysis using two databases: Laccase and Multicopper Oxidase Engineering Database (LccED, Swiss-Model Protein, respectively) showed the closest match is laccase and laccase-like enzymes [69]. We suspect that *N. stella* MCOs are laccase-like enzymes that may also be capable of oxidizing manganese, but this function must be addressed experimentally. Proteins responsible for Fe^2+^/Mn^2+^ uptake were expressed in all samples (Fig. 3E), indicating the capacity of *N. stella* to store intracellular iron and manganese, essential cofactors for various enzymes (e.g., superoxide dismutase, Fructose 1,6-bisphosphatase).

## Discussion

Our results demonstrate that these *N. stella* populations possess an impressive array of metabolic pathways (Table 2) enabling the utilization of both organic and inorganic compounds to sustain the flow of electrons and generate energy. These pathways potentially augment CO_2_ assimilation through the CBB cycle in the absence of light and oxygen. Thus, *N. stella* that live among the microbial mat of the euxinic SBB seafloor are examples of chemolithomixotrophic protists.

**Table 2:** Metabolic pathways performed by SBB *N. stella.*

Metabolism	Localization	Similarities	Novel to foraminifera	Novel to eukaryotes	Initial documentation in foraminifera
Dark carbon fixation via CBB	Kleptoplast	Bacteria, archaea	No	No	This study[11]
Fumarate reduction	Mitochondria	Anaerobic protist	No	No	[10]
Denitrification	Mitochondria and associated symbionts	Fungi and foraminifera	No	No	[23, 98]
Fermentation	Mitochondria and cytosol	Fungi and protist	No	No	[11, 98]
Proton-pumping pyrophosphatases (H + PPases)	Mitochondria	Plants	Yes	No	This study
Unconventional anaerobic respiration via H_2_O_2_ reduction	Mitochondria	Eukaryotes and fungi	Yes	Yes	This study
(PPK)-acetate kinase (Ack) pathway	Mitochondria and cytosol	Bacteria, archaea anaerobic protist, plants	Yes	Yes	This study
Cysteine synthase pathway	Mitochondria and cytosol	Bacteria, archaea and ciliates	Yes	No	This study
PEP-pyruvate-oxaloacetate	Mitochondrial and cytosol	Anaerobic protist and plants	Yes	No	This study
Pyrophosphate-dependent glucose metabolism	Mitochondria and cytosol	Bacteria, archaea and eukaryotes	Yes	No	This study
Sulfate reduction/assimilation	Mitochondria	Photosynthetic eukaryotes	No	No	This study
Sulfite oxidation as energy and electron donor	Mitochondria	Bacteria, archaea and ciliates	Yes	Yes	This study
Manganese oxidation and manganese/iron transporter	Cytosol and membrane-bound enzymes	Bacteria, archaea and fungi	Yes	No (only in fungi, refs, 87 and 88)	This study

### Inorganic carbon assimilation

The ability of foraminifera to sequester diatom chloroplasts and to retain the function of these chloroplasts for assimilating inorganic carbon has been reported in several species that inhabit shallow and intertidal photic zones [3, 7]. Although the SBB *N. stella* live far below the base of the euphotic zone and sequester *Skeletonema pseudocostatum* chloroplasts [4, 11], the full functionality of these organelles had not yet been demonstrated. This work reports the high expression of *rbcL* genes alongside chloroplast-related proteins (Fig. 1A), suggesting the likely functionality of these sequestered chloroplasts in inorganic carbon assimilation via CBB. Additionally, the expression of PSI, PSII and ATP synthase provides further evidence that the thylakoid membrane is intact and functional (Fig. 1A–D; Supplemental Fig. 1A).

The functionality of *N. stella*’*s* CBB pathway was brought into question, however, when our data revealed trends of two key genes: phosphoribulokinase (PRK), which was downregulated, and sedoheptulose-bisphosphatase (SBPase), whose gene expression was not detected. Previous studies showed that SBPase expression is not essential for the functionality of the CBB pathway in chemolithoautotrophic bacteria, where autotrophic CO_2_ fixation is mediated by transaldolase, substituting for SBPase in the CBB cycle [70]. Similarly, in cyanobacteria, the enzyme FBPase can perform a bifunctional role, hydrolyzing both FBP and SBP [71]. In the CBB cycle regeneration phase, both PRK and SBPase genes are not redox regulated in diatoms, unlike in plants [71]. A recent study that designed a mutant PRK gene and tested its function in the green algae *Chlamydomonas reinhardtii* showed that CBB activity was retained even if PRK expression was downregulated, with maximal photosynthetic activity retained at 86% PRK expression [72]. Furthermore, in many metazoans and protists with functional plastids, neither PRK nor SBPase were detected in genomic and transcriptome data, suggesting that their plastid functional stability is the result of long-term maintenance of cryptic algal proteins that persist months after acquiring the chloroplast from algal prey [73]. Additionally, in the green *N. stella* samples, the expression of plastid-targeting proteins such as CP12, cytochrome C6, and FCP [41, 74] (Fig. 1D) suggest that all proteins essential to an active CBB cycle are maintained. These findings suggest that *N. stella* is capable of fixing inorganic carbon in dark euxinic niches.

To assess if the CBB cycle is active, an in situ incubation, using H^13^CO_3_^−^ as substrate, was executed. Changes in δ^13^C values of the POC pool in surface sediments after the *in-situ* incubations using injector pushcores were minimal and comparable to sediment values with unlabeled seawater. These POC samples were acidified with concentrated acid (12 N HCl, 300 μl, overnight), but were not purged with He or N to physically displace inorganic carbon. Without purging, it is possible that ^13^CO_2_^−^ could adhere to surfaces or remain dissolved, however, given the small sample volumes (<1 ml) and acidic pH we expect the inorganic pool to move quantitatively to the gaseous CO_2_ pool. Of note, the *N. stella* tests are very thin, around 1–2 microns thick [75], and should dissolve readily during acidification. If this acidification was not successful, we would expect a small positive excursion in all measured isotopic values. Estimates of this excursion are +1 permil if 100 μl of incubation water (~5 mM HCO_3_^−^ at 50 atom%) adhere to the samples (i.e. remained through acidification). Rather, isotopic values from in situ incubated samples displayed a trend toward ^13^C enrichment up to 3x this estimate, consistent with some uptake of the ^13^C label over the 23-hour incubation. Because these in situ incubations included the whole community, rather than targeting only the foraminifera, these values are expected to underestimate uptake of labeled inorganic carbon by the foraminifera. This underestimate stems from the assumption that the >63-μm sediment fraction contains foraminifera plus other large contributors to the organic carbon pool (e.g., other large protists, metazoan meiofauna, detritus) that would not incorporate the label. For the δ^13^C of the POC pool to become more enriched requires the addition of ^13^C and if we assume only the foraminifera (specifically *N. stella*) were assimilating this carbon in the >63-μm size class then this net assimilation underestimates the foraminifera-specific assimilation rates. Another possibility is that labeled bicarbonate was incorporated by phagocytosis of bacterial or archaeal autotrophs by the foraminifera, although SBB *N. stella* typically does not phagocytose (Supplemental Fig. 1B and C; [11]) and these incubations were conducted in the absence of sediments. Prokaryotes could possibly have been carried over into the incubations because they remained stuck to the surface of the picked and filtered sterile seawater-washed foraminifera used for the incubations. Alternatively, the more positive δ^13^C values of the POC could be explained by preferential removal of ^12^C through respiration. We cannot exclude this possibility either. However, if we take together the δ^13^C isotopic values and the N_2_ production values, a trend exists between N_2_ production and more positive δ^13^C values. This is consistent with heterotrophic denitrification or coincident denitrification and inorganic carbon assimilation. Certain foraminifera are known to conduct complete denitrification through anaerobic and aerobic processes. N_2_O production, an intermediate produced within the denitrification pathway, was also observed, but did not show a trend with δ^13^C values. However, due to the heterogeneous nature of the sediment samples, it is impossible to verify that either the N_2_ production or the change in carbon isotopic values was exclusively due to *N. stella* activity. To shed light on the role of *N. stella* and its kleptoplasts in the CBB cycle, a more targeted incubation study was conducted to distinguish if the observed isotopic shift was due to a loss of ^12^C or an uptake of ^13^C.

*N. stella* isolated from and cleaned of SBB sediments were incubated in the laboratory with ^13^C-labeled bicarbonate and movement of the tracer into their biomass was tracked. Movement of the labeled ^13^C from the dissolved inorganic carbon (DIC) pool to the *N. stella* pool was consistent with either ^13^C exchange between DIC and POC (the foraminifera were measured after acidification treatment to remove inorganic material) or active carbon uptake into the *N. stella* cellular biomass. *N. stella* in the control treatments (HCO_3_^−^, sulfide and nitrate or ammonium but no ^13^C-label) showed isotope values consistent with marine organic carbon (δ^13^C ≈ −22‰) and not marine inorganic carbon (−5‰), consistent with effective removal of the inorganic shell material (and suggesting purging with He or N_2_ was not needed). If shell carbonate remained, it would push our isotopic data more positive (toward −5‰), a trend not observed in this data set (Table 1, Supplemental Fig. 6A). Similarly, the lack H^13^CO_3_^−^ removal due to the lack of purging with He/N_2_ in the acidification step of sample processing would shift measured values positive relative to values for marine organic carbon and at most by +1 permil (assuming 100 μl of incubation waters remained after filtering). We do not see this change between our no-isotope (^13^C) control and our nitrate amendments, which were not significantly different than the no-^13^C controls (Supplemental Fig. 6A). The consistency of the nitrate-amended sample values with the no-isotope control and marine organic material is consistent with the removal inorganic H^13^CO_3_^−^ prior to measurement (i.e. acidification was sufficient to remove DIC). In the presence of nitrate, *N. stella* biomass was enriched in ^13^C but enrichment was not significantly different from control incubations at this sampling effort, suggesting added nitrate did not stimulate prolonged carbon uptake. Ammonium amendments showed a more striking trend. With ammonium added to incubations, the δ^13^C of the *N. stella* biomass was about 3.2‰ enriched after 20 hour of incubation. These shifts in δ^13^C of the *N. stella* biomass can be placed in context by calculating the total additional carbon that would have to be added from the H^13^CO_3_^−^ pool to achieve these isotopic signatures. As described, *N. stella* has an average calculated biomass of 0.06 μg C/individual. To shift the isotopic signature of this organic pool in the ammonium amended incubations to the measured values would require between a ~ 0.13% and 0.5761% (Table 1) addition of biomass C in the nitrate and ammonium-amended incubations respectively, over a 20-hour incubation. This determination is without consideration of inorganic carbon in the foraminifera tests, as the tests were removed by acidification prior to analysis. The No Isotope Control foraminifera underwent the same sample preparation steps. However, this could be an underestimation of carbon uptake if non-active specimens (i.e. dead or dormant; [76]) were included, or if adhered detrital carbon associated cells were included in the incubation pool. The isotopic shift was similar between *N. stella* isolated using red light and white light, suggesting the limited exposure to potentially PAR was not a determinant in C-uptake. C-uptake was not as pronounced in nitrate amended incubations as in ammonium amended incubations, resulting in <1‰ shift in uptake even after 20 hours. This could be because ammonium was already reduced and more efficiently assimilated into biochemical pathways than nitrate. The combined evidence from metatranscriptomic analyses and the incubation studies suggest SBB *N. stella* incorporate inorganic carbon into biomass, likely using the CBB pathway; such abilities support the assertion that SBB *N. stella* have chemoautotrophic capabilities.

### Energy sources and oxygen-independent metabolism

We focused on identifying pathways in the metatranscriptome data for utilization of organic carbon and inorganic sulfur compounds to discern the strategy used by *N. stella* to generate energy and reducing equivalents (i.e. NADPH/NADH) from metabolites necessary for fixing CO_2_ in darkness and euxinia. Our findings revealed robust expression of genes involved in anaerobic oxidation of sulfide across all our samples. Although sulfide oxidation is a common feature in mitochondria across a wide range of organisms, from micro-eukaryotes to humans, hydrogen sulfide (H₂S) can meet energy demands during hypoxia and regulate electron flow in the electron transport chain. Specifically, H₂S may influence the reduction of malate to succinate, thereby facilitating ATP production under low-oxygen conditions [77, 78]. We hypothesize that sulfide oxidation plays a significant role as an electron donor to sustain electron flow in *N. stella*. These electrons can then be transferred by cytochrome C-type proteins across the chloroplast thylakoid membrane generating a pmf for the generation of ATP via kleptoplast ATP synthases, ultimately allowing for carbon fixation via CBB cycle (Fig. 2). Previous experimental studies reported that diatoms meet their energy needs for carbon assimilation through energy exchange from mitochondria to plastids [79]). Additionally, it is established that nucleotide transporters (NTTs) transfer ADP/ATP from the cytosol to the diatom’s plastids [80]. Also, the fact that photosystems I and II were active suggests that cyclic electron flow (CEF) can be an alternative pathway for translocating extra protons into the lumen [81].

It is unlikely that the *N. stella* in this study were performing aerobic respiration at the time of sample collection and fixation because the habitat sampled had undetectable oxygen and was highly sulfidic. Further, *N. stella* inconsistently expressed cytochrome c oxidase subunits across all pooled samples (Supplemental Fig. 8). Our data showed that *N. stella* have adopted several oxygen-independent carbon catabolic pathways (Fig. 2). The absence of pyruvate carboxylase, and the initial metabolic link of the TCA cycle (i.e. citrate synthase, [10]), collectively suggest that *N. stella*’s central carbon metabolism operates independently of the canonical TCA cycle and oxidative phosphorylation, which are the typical eukaryotic strategies for thriving in anoxic habitats [82]. *N. stella* has evolved metabolic pathways specialized for extended glycolysis metabolism and for metabolizing organic compounds, amino acids, and simple sugars. These pathways bear resemblance to those observed in an anaerobic flagellate protist [83, 84]. Our findings document the expressed anaerobic pyruvate-formate lyase and phosphoketolase (PPK) in a foraminifer (Figs 2 and 3B). Phosphoketolase expression noted because this enzyme has the potential to enhance carbon flux, preventing carbon loss [55] by producing high energy acetyl-phosphate and G3P or E4P from any phosphate sugar manufactured by the phosphate pentose pathway (PPP) or CBB cycle [85]. This ability is thought to be unique to bacteria, algae, and some fungi; but it appears that foraminifera as well can grow on pentose sugar (i.e. xylose) as a carbon source [86]. Moreover, SBB *N. stella* can use the supply of G3P in the penultimate step of glycolysis to create additional ATP and NADPH.

Micro-eukaryotes with a reduced mitochondrial genome, adapted for an anaerobic lifestyle, usually rely on extended glycolysis and fermentation for ATP production [84, 87, 88]. *N. stella* from SBB does things differently. Specifically, *N. stella* expresses the typical pyruvate kinase (PK) as well as an uncommon enzyme, pyruvate phosphate dikinase (PPDK), and it uses inorganic pyrophosphate (PPi) to convert phosphoenolpyruvate (PEP) to pyruvate. Together with PPi-PFK, these enzymes conserve ATP consumption. This allows the generated ATP from glycolysis and fermentation to be utilized for more efficient cellular functions. PPDK has solely been identified in a few anaerobic protists, offering a selective evolutionary advantage to those lacking aerobic mitochondria [84, 89]. PPDK is usually localized to the cytosol or chloroplast in C4 plants [87, 90]. The PPDK in *N. stella* is, however, uniquely localized to mitochondria (Supplemental Table 1). It appears that *N. stella* has metabolic pathways to augment their pyruvate flux (Fig. 2). Malate produced from the peroxisomal glyoxylate cycle [10] can be converted it into pyruvate through malic enzyme (ME), or to OAA and then pyruvate by malate dehydrogenase and oadA, respectively (Fig. 2). The utilization of oxygen-independent pyruvate metabolism with PFL and the previously reported PFOR [10, 11] also highlight that the central route for ATP and acetyl-CoA generation in *N. stella* is consistently specialized to anoxia/euxinia. These results show the evolutionary convergence between the metabolism of *N. stella* and fungal, bacterial, and archaeal metabolisms [91].

### Inorganic pyrophosphate metabolism

Membrane-bound H^+^-pyrophosphatases (H^+^-PPases) generate a pmf by hydrolyzing inorganic pyrophosphate (PPi), a high-energy metabolite that potentially supports ATP production (see Figs 2 and 3A) [69]. Although H^+^-PPases have been previously identified in various protist species [92–94], our study reports their presence in foraminifera. In addition to hydrolyzing PPi, H^+^-PPases can also synthesize PPi, which can serve as an energy source for several glycolytic metabolic pathways.

Energy production through PPi hydrolysis is considered the first energy source in the origin of life [92, 95, 96] and is used as an alternative or additional energy source when ATP production through ATPase synthase is low, although this point is debated [96]. Recent studies showed that certain benthic foraminifera store high concentrations of inorganic phosphate intracellularly [97]. The ability of benthic foraminifera to hydrolyze organic creatine phosphate was reported [98]. Here we show that foraminifera can utilize inorganic pyrophosphate via H^+^-PPases to maintain the mitochondrial proton gradient (Figs 2 and 3A). These findings shed light on the key role of synthesis and hydrolysis of inorganic pyrophosphate through the H^+^-PPase in *N. stella,* and the likely importance of this as an evolutionary adaptation for generating and conserving energy in an anoxic realm.

We also observed that SBB *N. stella* express PPi-dependent glycolysis using PPi-PFK, PPi-PPDK, and, possibly, the PPi- dependent sulfate reduction pathway, indicating PPi is an energy substitute to ATP, a unique evolutionary trait only known to date in bacteria and archaea, anoxia-tolerant plants, and a few amitochondriate anaerobic protists [46, 99, 100]. The utilization of PPi-dependent metabolism as an evolutionary strategy to meet the cell’s energy demands when oxygen is scarce or absent is now expanding to include this foraminifer that thrives in anoxic, sulfidic seafloor sediments.

### Non-chloroplastidic sulfate assimilation

A recent correlative TEM-NanoSIMS (Nanoscale Secondary Ion Mass Spectrometry) study of kleptoplastidic and non-kleptoplastidic benthic foraminifera species from varied marine environments showed their ability to uptake inorganic nitrogen (ammonium or nitrate) and sulfate into their biomass [101], suggesting an assimilatory function. We identified a sulfate assimilation pathway in *N. stella* and our amino acid sequence analysis suggests that sulfate assimilation is localized to the mitochondria, independent from kleptoplasts. In photosynthetic eukaryotes, sulfate assimilation is localized to the chloroplasts, whereas it is cytosolic in non-photosynthetic eukaryotes [102]. The chloroplastidic protist *Euglena gracilis* is the only known exception, where the pathway is localized to mitochondria [102], suggesting that sulfate assimilation in eukaryotes is not strictly associated with chloroplasts. Thus, the SBB *N. stella* is the second known chloroplastidic eukaryote to localize ammonium and sulfate assimilation in their mitochondria.

### Putative genes involved in redox reactions

In SBB, *N. stella* thrives within a sulfur-oxidizing microbial mat where pore waters can be rich in hydrogen peroxide. *N. stella*’s superoxide dismutase, an enzyme that scavenges superoxide radicals to produce oxygen and hydrogen peroxide, were highly expressed (Supplemental Fig. 9), consistent with prior observations [10]. It was hypothesized that SBB *N. stella* and some other foraminiferal species use catalase to cleave H_2_O_2_ to provide O_2_ to support aerobic respiration [103]. *N. stella* possesses and expresses an ROS defense system, the eukaryotic catalase/peroxidase (KatG/CAT) [10]. This is an efficient enzyme that protects against high (up to millimolar) concentrations of H_2_O_2_ by cleaving H_2_O_2_ into water and O_2_ [53]. Hydrogen peroxide metabolized by catalase (KatG/CAT; Supplemental Fig. 9) can be utilized for beta-oxidation in peroxisomes, assuming that catalases are situated near the peroxisomes [10]. However, we also detected the expression of the mitochondrial heme-containing cytochrome C peroxidase (Ccp) in our data across all samples (Figs 2 and 3A); this enzyme can contribute to H_2_O_2_ metabolism, but its exact function requires dedicated experimental study. Ccp functions differently in eukaryotes and bacteria. For example, in fungal mitochondria, the Ccp catalyzes H_2_O_2_ reduction, and its expression increases during aerobic respiration to protect the cells against oxidative stress [104]. *N. stella* expresses Ccp under euxinic environmental conditions and is not likely performing aerobic respiration. An experimental study identified Ccp as a respiratory oxidase that uses H_2_O_2_ as a terminal electron acceptor in *E. coli* when oxygen is not available [48]. Furthermore, the same study showed that the activity of Ccp and fumarate reductase (QFR) were comparable, where QFR used fumarate as a terminal electron acceptor and contributed to increased H_2_O_2_ production, suggesting both enzymes may be co-regulated to maintain cellular redox balance [48]. An experimental study linked an increased concentration of H_2_O_2_ to high concentration of ATP in *N. stella* and a coexisting foraminifer with copious peroxisomes (*Buliminella tenuata)* [103]. Based on our observations, H_2_O_2_ may be an alternative respiratory substrate besides nitrate in SBB *N. stella*, however, additional experimental evidence is required to resolve this possibility.

To date, the presence of multicopper oxidases identified as manganese oxidases and laccases have been only reported in fungi and bacteria, with few of those studies verifying the function experimentally [69, 105]. The fungal laccase belongs to MCO and works together with manganese peroxidases to oxidize Mn II to Mn IV to degrade the lignocellulose and yield H_2_O_2_ [106]. The expression of candidate 2d MCO genes with amino acid sequence similarity to laccase and laccase-like manganese oxidases in *N. stella* suggests they may play roles in metal oxidation (Figs 2 and 3E). It is unclear if *N. stella*’s MCOs perform similar functions as in basidiomycete fungi, where Mn (II) oxidase and peroxidase cooperate in degradation of lignin and xenobiotics to produce Mn (III) and H_2_O_2_ [107]. A benthic foraminifera species from Antarctica was able to uptake dissolved organic matter [108], however, those enzymatic and biochemical processes were not identified. We hypothesize that SBB *N. stella* are using their laccase-like enzyme for degradation and oxidation of organic matter. Experimental verification is required to test this hypothesis. Additionally, it is thought that *N. stella* and other foraminifers in oxygen-depleted environments incorporate soluble Mn (II) into their calcium carbonate test or shell in a ratio with calcium that is proportional to the in situ seawater oxygen concentration (e.g. [75, 109, 110]), thus allowing Mn/Ca to serve as a proxy for reconstructing past dissolved oxygen concentrations. Unfortunately, the SBB *N. stella* Mn/Ca did not fit expectations, revealing lower than predicted Mn/Ca ratios [75]. This observation, together with the presence of MCO enzymes, suggests *N. stella* may be utilizing Mn (II) for other biochemical processes, for instance, to maintain the redox potential of the chloroplast, to reduce superoxide or nitrate to provide electrons for various biochemical processes including denitrification and/or, possibly, carbon fixation. The fact that *N. stella* has abundant peroxisomes (Supplemental Fig. 1B and C; [6]) and all samples had high expression of superoxide dismutase (SOD) suggests that oxygen made by SOD can be used for manganese oxidation without being constrained by catalase activity [111]. To understand the link between Mn (II), carbon fixation, and denitrification, incubation experiments are required to provide evidence that *N. stella* can perform dark-driven manganese oxidation.

In conclusion, we show that in situ-preserved *N. stella*, a population that lives in aphotic (dark) anoxic to sulfidic sediments of the SBB, relies on oxygen-independent carbon metabolism and uses both organic and inorganic (sulfide and Mn II) substrates as energy and electron sources. The ability of *N. stella* to retain functional chloroplasts to fix carbon via the CBB cycle is supported by both gene expression and isotope analysis. Our findings support the assertion that *N. stella* is a micro-eukaryote with metabolic pathways to support chemolithomixotrophy. The versatility of this kleptoplastidic foraminifera and its unique mitochondria metabolism represents an evolutionary puzzle that should change how we perceive the origin and diversification of eukaryotes on Earth and bolster the need to study dark euxinic habitats elsewhere.

## Supplementary Material

Supplemental_materials_Jan14_2025_Final_wrae248

Supp_Table1_stella_prediction_unclustered_wrae248

## Data Availability

All data presented in this manuscript have been submitted to the NCBI under the BioProject submission number PRJNA1158755. The raw transcriptome and DNA sequencing reads are deposited in the NCBI repository (SRX26353516 to SRX26353525). Additionally, the final processed files, including each transcriptome’s assembled sequences, predicted functions, and sample-specific abundances, have been uploaded to Figshare (DOI: 10.6084/m9.figshare.26969926).
