# Supplementary material for: Array of metabolic pathways in a kleptoplastidic foraminiferan protist supports chemoautotrophy in dark, euxinic seafloor sediments
Source: ISME J. 2024 Dec 13;19(1):wrae248. doi: 10.1093/ismejo/wrae248 (PMC11736642; doi:10.1093/ismejo/wrae248)
Supplement: Supplemental_materials_Jan14_2025_Final_wrae248 [file supplemental_materials_jan14_2025_final_wrae248.pdf]

Array of metabolic pathways in a kleptoplastidic foraminiferan protist supports chemoautotrophy in dark, euxinic seafloor sediments

Fatma Gomaa[1,2*], Daniel R. Rogers[3], Daniel R. Utter[4], Christopher Powers[5], I-ting Huang[2], David J. Beaudoin[6], Ying Zhang[5], Colleen Cavanaugh[2], Virginia P. Edgcomb[1], Joan M. Bernhard[1*]

[1] Department of Geology and Geophysics, Woods Hole Oceanographic Institution, Woods Hole, MA, USA; fgomaa@whoi.edu; vedgcomb@whoi.edu; jbernhard@whoi.edu,

[2] Department of Organismic and Evolutionary Biology, Harvard University, Cambridge, MA 02138, USA; fatma.gomaa@gmail.com; cavanaug@fas.harvard.edu, ihuang@g.harvard.edu

[3] Chemistry Department, Stonehill College, Easton, USA; drogers2@stonehill.edu

[4] Division of Geological and Planetary Sciences, California Institute of Technology, Pasadena, CA 91125, USA; dutter@caltech.edu

[5] Department of Cell and Molecular Biology, College of the Environment and Life Sciences, University of Rhode Island, Kingston, RI, USA; c-11060@uri.edu, yingzhang@uri.edu

[6] Department of Biology, Woods Hole Oceanographic Institution, Woods Hole, MA, USA; dbeaudoin@whoi.edu

* Corresponding authors: Fatma Gomaa and Joan M. Bernhard

Supplemental Figures

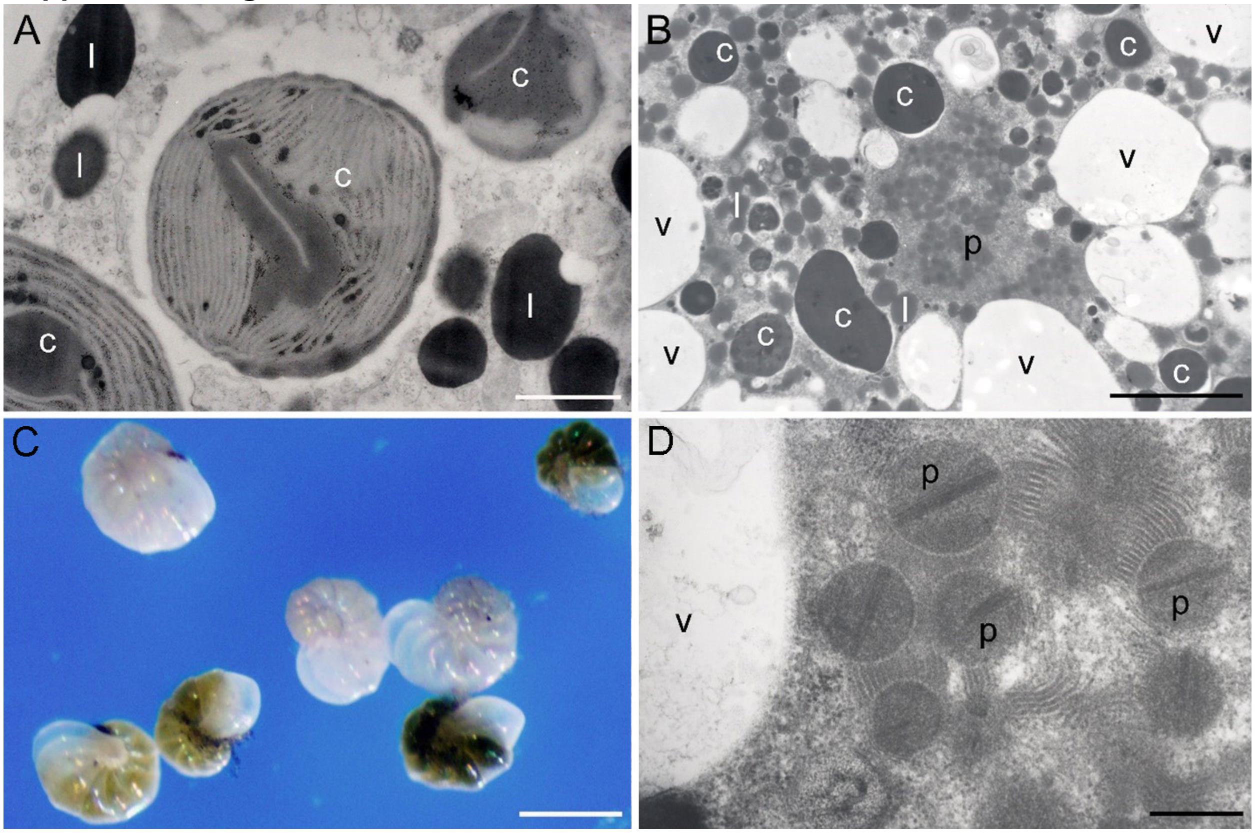

Supplemental Figure 1. *Nonionella stella* from Santa Barbara Basin. **A, B, D.** TEM micrographs. **A.** sequestered chloroplasts (c) with lipids (l). **B.** Overview showing chloroplasts, lipids, "empty" vacuoles (v) and peroxisomes (p). **C.** Reflected-light micrograph showing small pool (n= 7) of partially cleaned specimens, revealing different cytoplasmic colors (4 green; 3 pink; white not shown). **D.** Clump of peroxisomes complexed with endoplasmic reticulum; vacuole also shown. Scales: A = 1 μm, B = 5 μm, C = 200 μm, D = 0.5 μm.

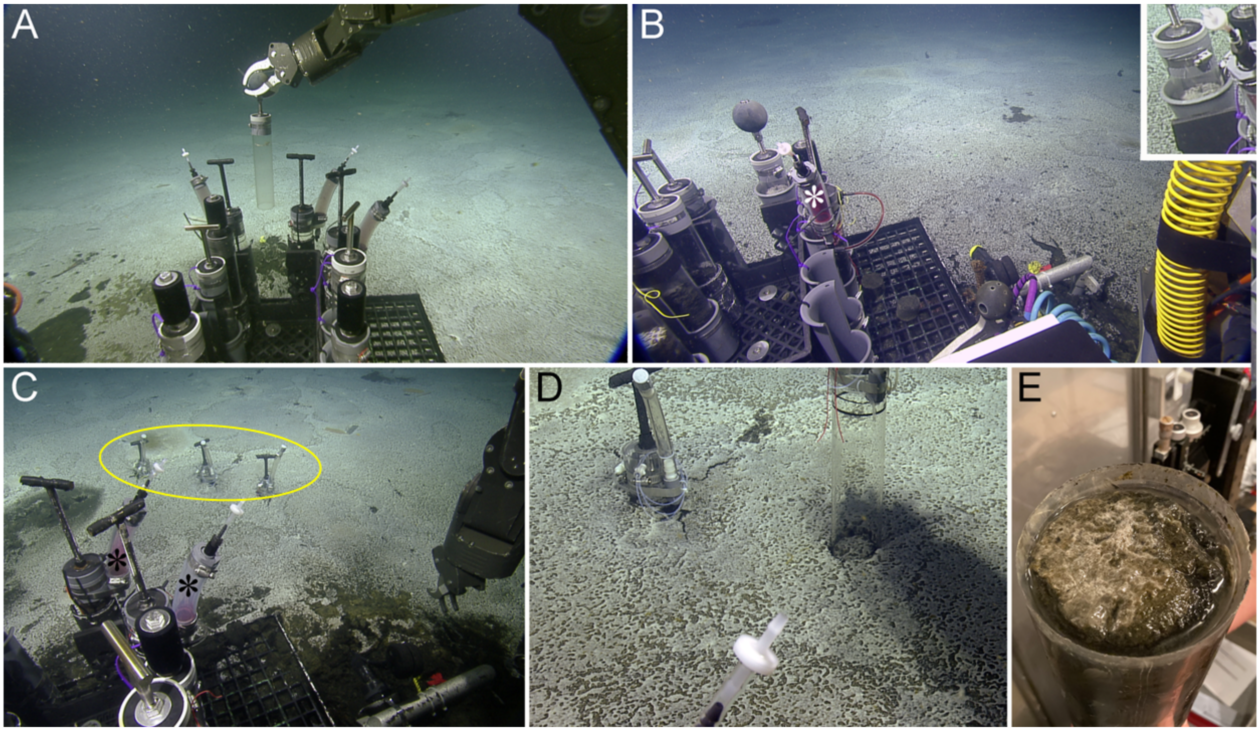

Supplemental Figure 2. **A-D**. Photographs of ROV *Hercules* coring operations showing extensive well developed white microbial mat on the sediment surface of SBB, 14 July 2021 between 571-572-m water depth (dives H1851 and H1852). **A**. ROV manipulator holding empty pushcore. **B.** View showing pushcore loaded with RNAlater loaded into large-diameter injector (*, cylinder filled with dark pink liquid) and a "regular" pushcore with intact sediment-water interface (inset). **C.** View showing two RNAlater pushcores in quivers (i.e., already injected/preserved; *) and three pushcores emplaced in seafloor for *in situ* $H^{13}CO_3$ incubations (yellow oval). **D.** Two of the *in-situ* bicarbonate incubation pushcores with the one on the left already emplaced and the one on the right being inserted into the seafloor. **E.** Multicore surface with white microbial mat, after bottom water was removed; collected on 8 July 2022 from 579-m water depth (cruise SP2213 event G). **Photo credits: A-D, Ocean Exploration Trust, NOAA Ocean Exploration, and NASA; E. Johana Rotterova, Univ Rhode Island.**

In situ fixation (ROV injector pushcores with RNAlater)
- 5 green *N. stella* pools (on ice)
- 2 pink *N. stella* pools (on ice)
- 1 white *N stella* pools (on ice)
- Cleaned (chilled SFSW) and transferred to DNA/RNA Shield
- @ WHOI: (meta)transcriptomes
- (holobiont: host+symbionts)

Live specimens (ROV pushcores)
- Surface ~2 cm sediment in bottom waters (no header space)
- @ WHOI, isolated on ice
- 2 green *N. stella* pools isolated
- (n = 715, 110)
- Cleaned (SFSW)
- MAGs analyses

In situ incubation (ROV injector pushcores with ^{13}C-label, ~24 hr)
- Processed at sea with red light
- Bicarbonate uptake
- $\delta^{13}C$-POC measured
- N_2 production measured

Live specimens (Soutar boxcores)
- Surface ~2 cm sediment in bottom waters (no header space), taken to WHOI
- Live green *N. stella* pools isolated (n= 10 individuals/ incubation), cleaned (SFSW)
- 20-hr incubations with sulfide+ $H^{13}CO_3^-$, with NH_4^+, $+NO_3^-$ or no N; controls (no isotope, but with sulfide)

Supplemental Figure 3. Workflow chart detailing the four distinct experiments conducted in our study.

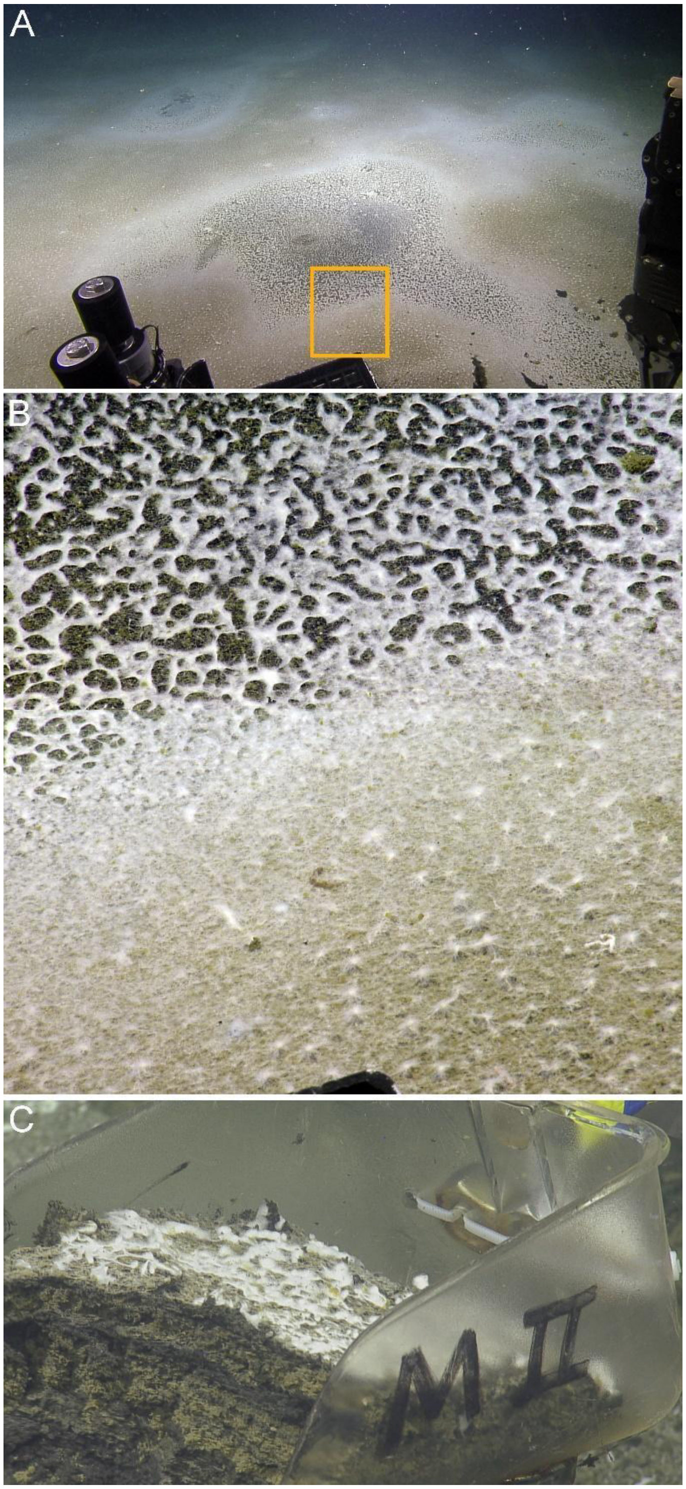

Supplemental Figure 4. A. Overview of extensive white microbial mat observed at 580-m water depth on dive H1850 (13 July 2021). **B.** Close up view of bacterial mat (orange box in A) showing gradational developmental differences. **C**. Scoop of SBB laminated sediments showing microbial mat surface and ~mm-scale laminae. Width of scoop is ~15 cm (taken on 15 July, H1855, 571-m water depth). **Photo credits: Ocean Exploration Trust, NOAA Ocean Exploration, and NASA.**

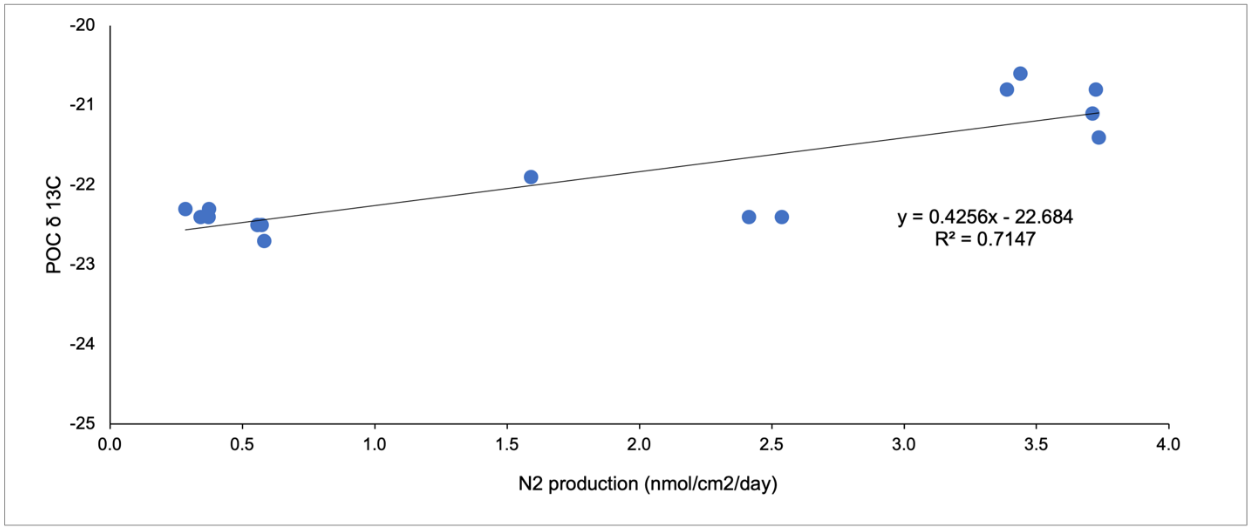

Supplemental Figure 5. Results from *in situ* incubations conducted in core liners at the seafloor show both the net production of N_2 and drawdown of $H^{13}CO_3$. There is a positive trend with greater $H^{13}CO_3$ uptake coinciding with greater net production of N_2. No trend was observed between net N_2O production and $\delta^{13}C$ of the POC pool (data not shown). Incubations were performed on the seafloor over 23 hr and amended with ~50 atom % $H^{13}CO_3$ (~5 mM).

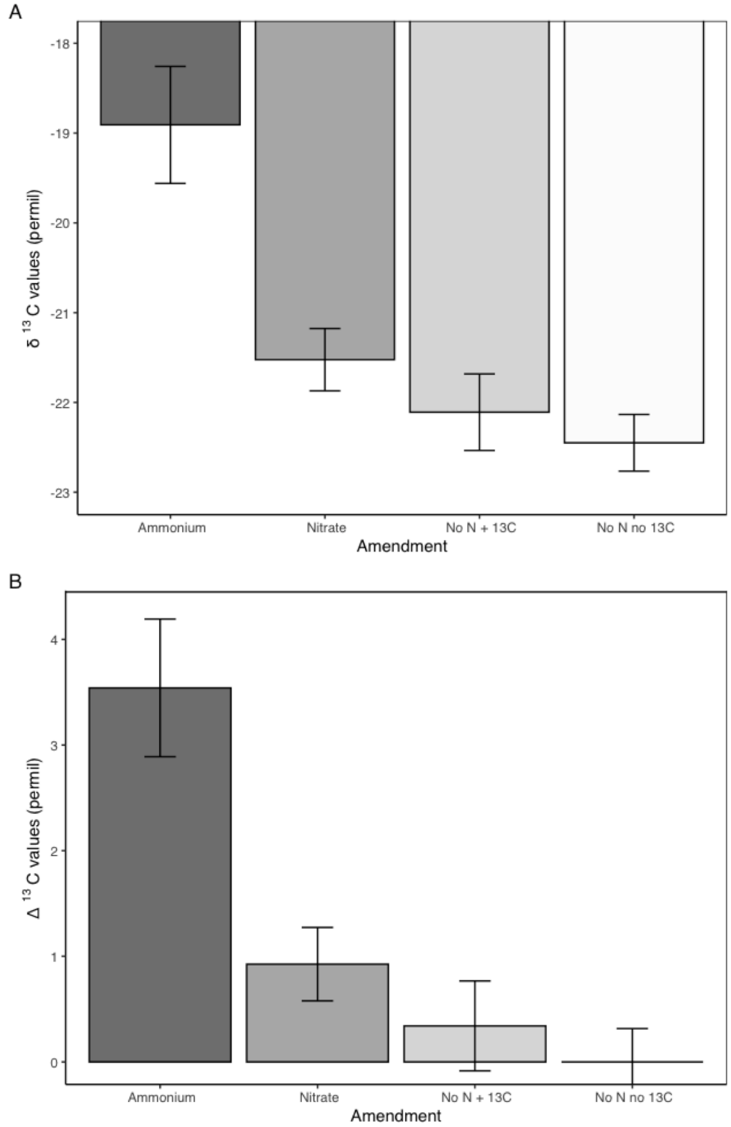

Supplemental Figure 6. Isotopic shifts of N. stella (n=10/incubation) amended with ^{13}C-bicarbonate (2 mM $H^{13}CO_3^-$), sulfide (2 µM), and nitrate (100 µM, n=5), ammonium (50 µM, n=5) or no nitrogen source (n=2). No isotope control incubations (n=2) were done without the ^{13}C label. All incubations were done under dark conditions and at environmental temperatures (6°C). A. After 20 hrs. of incubation, the ammonium additions (n=5) resulted in significant ($p<0.10$) ^{13}C incorporation into biomass relative to the no isotope control. Nitrate addition also show a trend toward enriched POC but statistical significance is limited by sampling effort. B. Data normalized to the isotopic signature of the no isotope control samples ($\Delta^{13}C$) shows a significant (~3.2 permil) enrichment in ^{13}C in the ammonium treatment.

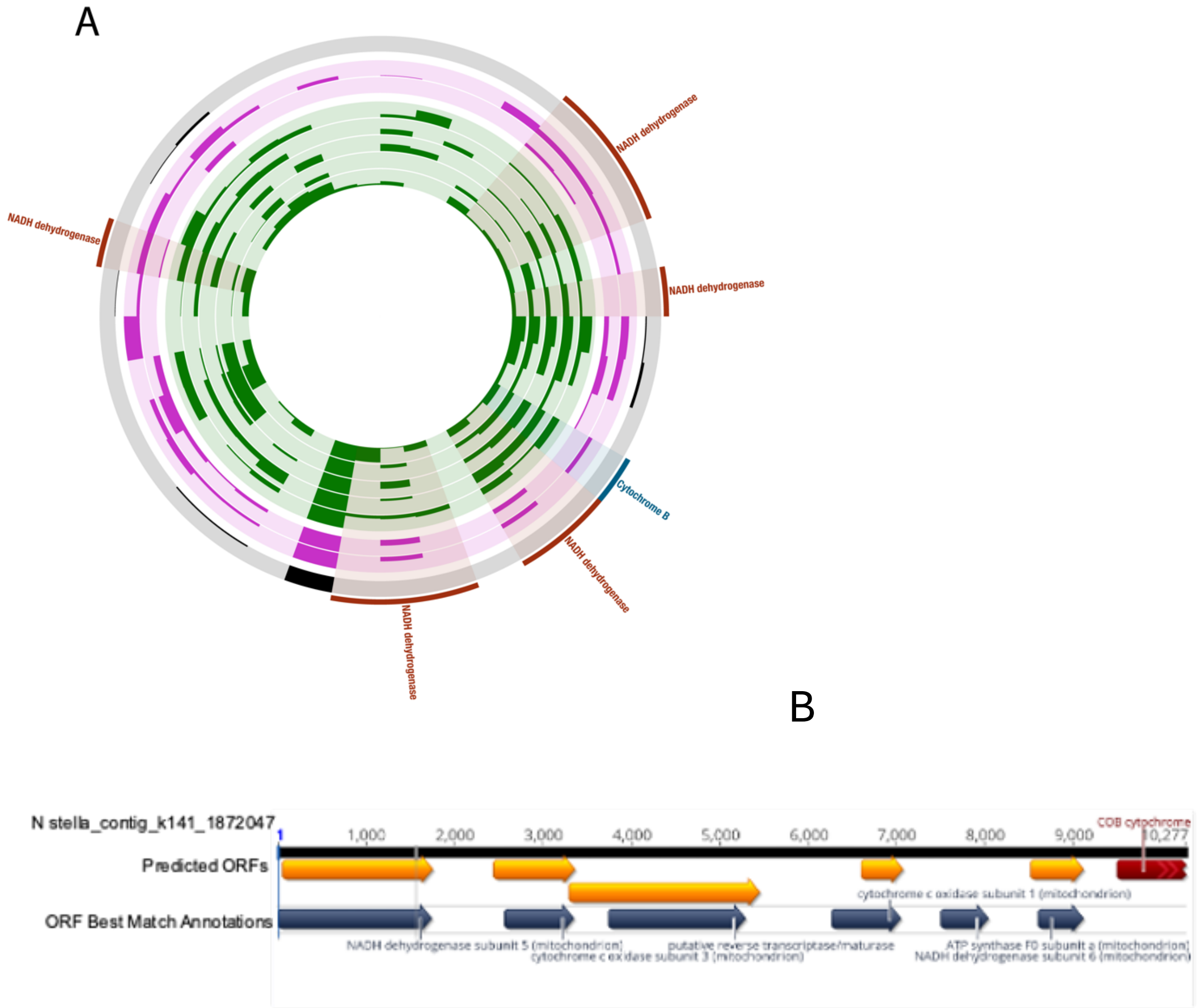

Supplemental Figure 7. A: Expression of diatom mitochondrial genes from SBB *N. stella* metatranscriptome reads. RNA-reads of *N. stella* mapped onto the mitochondrial genome of the diatom *Skeletonema pseudocostatum*. Rings represent the green-colored *N. stella* samples (n=5), the pink-colored *N. stella* (n=2) samples, and the white-colored *N. stella* sample. Expression of cytochrome B (*cob*) gene is displayed in the green *N. stella* samples and one of the pink samples. Height of green, pink or black solid color in each ring represents the maximum detection of each gene in the green, pink and the white *N. stella*. Note sparse and fragmented mapping of diatom's mitochondrial genes retrieved from *N. stella*. **B:** MAG analyses showing that the (cytochrome B) *cob* gene is encoded in a 10 kb contig that comprises NADH dehydrogenase subunit. ORFs in the contig are assigned to diatom. Yellow arrows indicate predicted ORFs (predicted by ORF Finder), dark Blue arrows indicate annotations with high sequence similarity as identified from the NCBI reference database, aligned by DIAMOND [104]. ORFs are represented by arrows, the directionality and the position of the arrows indicates ORF position and orientation.

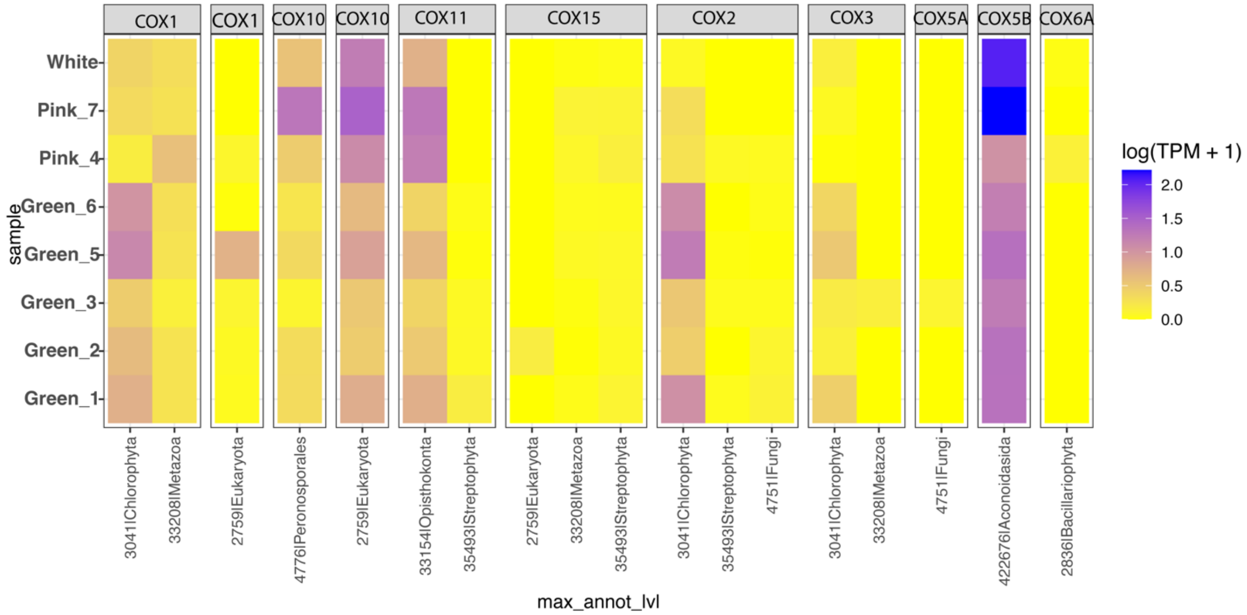

Supplemental Figure 8. Heatmap showing the expression of all subunits of the Cytochrome C Oxidase (O_2 respiratory electron transport chain IV), the expression of COX3, 4, 6A, 6B, 6C, 7, 8, 15, 17 subunits were inconsistent or not detected among the different types of *N. stella* samples, the annotation on x-axis is at the class-level.

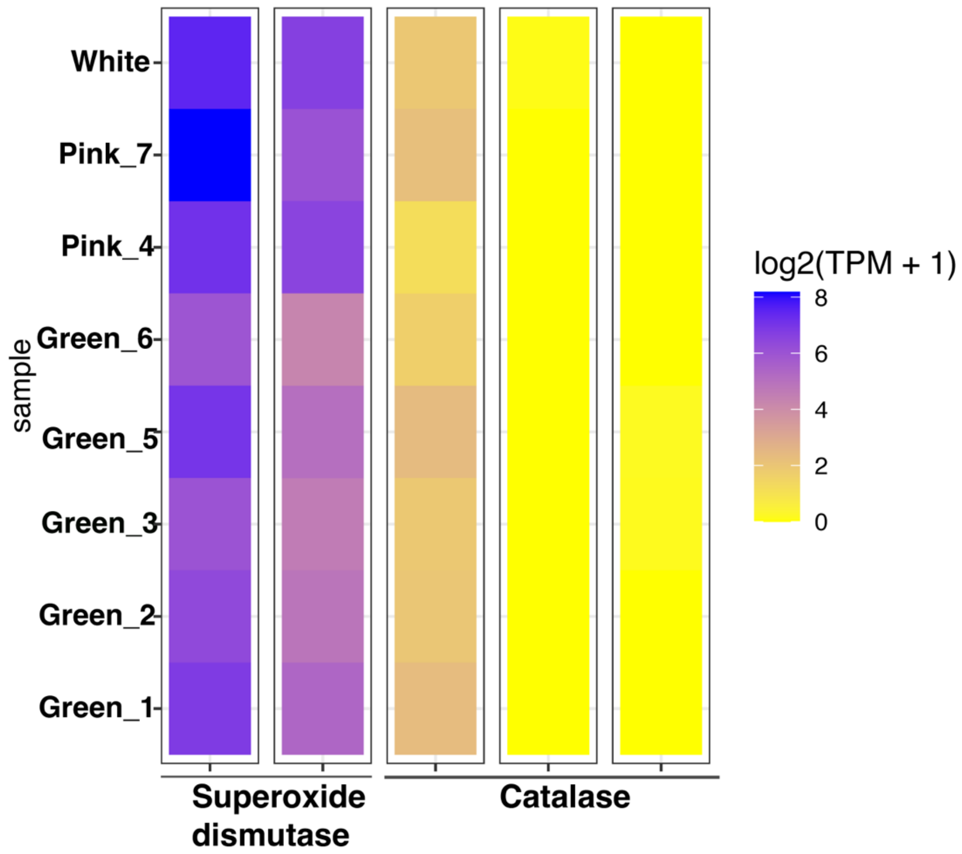

Supplemental Figure 9. Expression profiles of Catalase (Kat) and superoxide dismutase (SOD) isoforms across all *N. stella* samples, showing that SOD is highly expressed across all samples compared to Kat expression. The fold increase is about 800 times**.**
